# Whole genome sequencing of *Trypanosoma cruzi* field isolates reveals extensive genomic variability and complex aneuploidy patterns within TcII DTU

**DOI:** 10.1186/s12864-018-5198-4

**Published:** 2018-11-13

**Authors:** João Luís Reis-Cunha, Rodrigo P. Baptista, Gabriela F. Rodrigues-Luiz, Anderson Coqueiro-dos-Santos, Hugo O. Valdivia, Laila Viana de Almeida, Mariana Santos Cardoso, Daniella Alchaar D’Ávila, Fernando Hugo Cunha Dias, Ricardo Toshio Fujiwara, Lúcia M. C. Galvão, Egler Chiari, Gustavo Coutinho Cerqueira, Daniella C. Bartholomeu

**Affiliations:** 1 Departamento de Parasitologia, Universidade Federal de Minas Gerais, Belo Horizonte, Brazil; 2 The University of Georgia, Athens, USA; 3 Departamento de Microbiologia, Imunologia e Parasitologia, Universidade Federal de Santa Catarina, Florianópolis, Brazil; 4 U.S. Naval Medical Research, Lima, Peru; 5 Broad Institute of MIT and Harvard, Boston, USA

**Keywords:** *Trypanosoma cruzi*, TcII, Field isolates, Genomic variability, Copy number variation, Ploidy

## Abstract

**Background:**

*Trypanosoma cruzi*, the etiologic agent of Chagas disease, is currently divided into six discrete typing units (DTUs), named TcI-TcVI. TcII is among the major DTUs enrolled in human infections in South America southern cone, where it is associated with severe cardiac and digestive symptoms. Despite the importance of TcII in Chagas disease epidemiology and pathology, so far, no genome-wide comparisons of the mitochondrial and nuclear genomes of TcII field isolates have been performed to track the variability and evolution of this DTU in endemic regions.

**Results:**

In the present work, we have sequenced and compared the whole nuclear and mitochondrial genomes of seven TcII strains isolated from chagasic patients from the central and northeastern regions of Minas Gerais, Brazil, revealing an extensive genetic variability within this DTU. A comparison of the phylogeny based on the nuclear or mitochondrial genomes revealed that the majority of branches were shared by both sequences. The subtle divergences in the branches are probably consequence of mitochondrial introgression events between TcII strains. Two *T. cruzi* strains isolated from patients living in the central region of Minas Gerais, S15 and S162a, were clustered in the nuclear and mitochondrial phylogeny analysis. These two strains were isolated from the other five by the Espinhaço Mountains, a geographic barrier that could have restricted the traffic of insect vectors during *T. cruzi* evolution in the Minas Gerais state. Finally, the presence of aneuploidies was evaluated, revealing that all seven TcII strains have a different pattern of chromosomal duplication/loss.

**Conclusions:**

Analysis of genomic variability and aneuploidies suggests that there is significant genomic variability within Minas Gerais TcII strains, which could be exploited by the parasite to allow rapid selection of favorable phenotypes. Also, the aneuploidy patterns vary among *T. cruzi* strains and does not correlate with the nuclear phylogeny, suggesting that chromosomal duplication/loss are recent and frequent events in the parasite evolution.

**Electronic supplementary material:**

The online version of this article (10.1186/s12864-018-5198-4) contains supplementary material, which is available to authorized users.

## Background

The protozoan parasite *Trypanosoma cruzi* is the etiologic agent of Chagas disease, a chronic debilitating illness that is endemic in Latin America, affecting ~ 5–8 million people and accounting for 662,000 disability adjusted life years [1–3]. Due to its extreme genomic and phenotypic variability, *T. cruzi* is currently divided into six discrete typing units (DTUs), named TcI – TcVI [4, 5]. The inclusion of a new DTU, Tcbat, which comprises bat-restricted trypanosomes, is still under debate [5, 6]. From the six DTUs, TcI, TcII, TcV and TcVI are usually involved with the domestic cycle of Chagas disease, accounting for the majority of the human infections [5]. Human infections caused by TcI strains are more prevalent from Central America to Bolivia, while TcII, TcV and TcVI human infections are more common in the Southern cone of South America, encompassing countries as Argentina, Chile, Paraguay, Bolivia and Brazil [5, 7–9]. Recently, several TcVI parasites were isolated from humans and vectors in Colombia, suggesting that the distribution of this DTU could be broader than previously speculated [10]. Although the division in six DTUs is well accepted, there are four major proposed models to explain *T. cruzi* evolutionary history [11–14]. Even though these models disagree about the ancestral strains and the number of hybridization events during *T. cruzi* evolution, they all agree that TcV and TcVI are hybrids, originated from parental TcII and TcIII strains. It is still unknown if these hybrids arose from a single hybridization [15] or from multiple independent recombination events [14, 16, 17]. Molecular dating suggests that these two hybrid lineages evolved recently; reinforcing the assumption that genetic exchange could still be driving the emergence of *T. cruzi* recombinant isolates [15, 16].

The prevalent hypothesis that *T. cruzi* replication is mostly clonal [18–20] is been confronted by several recent findings suggesting that recombination events are frequent in *T. cruzi* populations from close geographic regions [14, 21–24]. The majority of field evidences suggest that recombination in *T. cruzi* is a non-obligatory, but common feature, and that parasexual mechanisms could be involved in genetic exchange processes in this parasite [16, 17, 21, 25, 26]. In fact, the presence of Chromosomal Copy Number Variation (CCNV), the duplication or loss of whole chromosome sequences, in *T. cruzi* [27, 28] could be a result of a fusion of diploid parasite cells followed by genome erosion, in a similar way as the *Candida albicans* parasexual cycle [17, 29–31]. According to this model, during the mammalian stage of the infection, the nucleus of two parasite cells fuse, resulting in a polyploid progeny, which may lose some supernumerary chromosomes resulting in different degrees of chromosomal aneuploidies [17, 32]. This assumption is further supported by the subtetraploidy found in *T. cruzi* experimental hybrids [23, 30], and by the ~ 70% higher DNA content in hybrids when compared to parental strains [33, 34]. Recent experiments based on tiling arrays [27], or whole genome sequencing [28], have showed that CCNV vary among and even within *T. cruzi* DTUs, suggesting that aneuploidy is a common feature in this parasite. However, the extent of chromosomal variation in close-related field isolates, or even the rate in which these chromosomal duplication/deletion events occur in *T. cruzi* is still unknown.

*T. cruzi* belongs to Kinetoplastida order, which is characterized by having the mitochondrial genome composed by ~ 30 copies of 20-50 kb maxicircles and thousands of copies of ~ 1 kb minicircles, which together comprise the kinetoplast DNA (kDNA) [26, 35]. Maxicircles are the functional equivalent of eukaryotic mitochondrial DNA, encoding genes that in these parasites can be edited by the RNA editing machinery that lead to U-insertions/deletions directed by minicircle sequences to correct frameshifts and premature stop codons [36–39]. Minicircle sequences are highly heterogeneous even within a single clone [40], while maxicircle sequences are relatively homogeneous, at least within their coding regions which represent ~ 63% of the *T. cruzi* maxicircle genome [26]. Phylogenetic analyses of *T. cruzi* maxicircles lead to the identification of three mitochondrial clades: clade A comprising TcI maxicircles; clade B comprising TcIII, TcIV, TcV and TcVI maxicircles; and clade C comprising TcII maxicircles [11, 26]. Although maxicircle sequences are usually conserved within a DTU, several studies have showed evidences of intra-lineage mitochondrial introgression, where the maxicircle genome from a DTU is associated with a non-recombinant nuclear genome of a different DTU [11, 17, 25, 26, 41]. Besides introgression, the occurrence of minor heteroplasmy, a presence of heterogeneous mitochondrial genomes in an individual cell, has also been reported in the *T. cruzi* Sylvio X10 strain [17, 26]. The mechanism behind both processes as well as their importance to *T. cruzi* evolution is still unknown, however these processes could be important to satisfy the necessity to escape Muller’s ratchet, the irreversible accumulation of deleterious mutations resulting from clonal reproduction [17, 23].

The majority of TcII infections occur in South American countries, such as Brazil and Argentina, being responsible for severe acute infections and by chronical mixed symptomatology with megaesophagus/megacolon and chagasic cardiomegaly [5, 11, 42]. Despite the importance of TcII in Chagas disease epidemiology and pathology, so far, no genome-wide comparisons of TcII field isolates have been performed to track the variability and evolution of this DTU in endemic regions. In the present work, we have sequenced the whole nuclear and mitochondrial genomes of seven TcII strains, which were recently isolated from chagasic patients with the indeterminate or cardiac forms of Chagas disease. These isolates where originated from the central and northeastern regions of Minas Gerais state, Brazil, an endemic region for *T. cruzi* TcII infection. We evaluated and compared the phylogeny of these TcII field isolates, as well as from strains from the TcI, TcII, TcIII and TcVI DTUs, based on nuclear and mitochondrial conserved genes to identify correlations among geographic and phylogenetic data. We also used SNP calling and Read Depth Coverage (RDC) analysis to estimate nuclear CCNV and mitochondrial heteroplasmy, revealing large divergences among TcII field isolates.

## Results

**Fig. 1:**
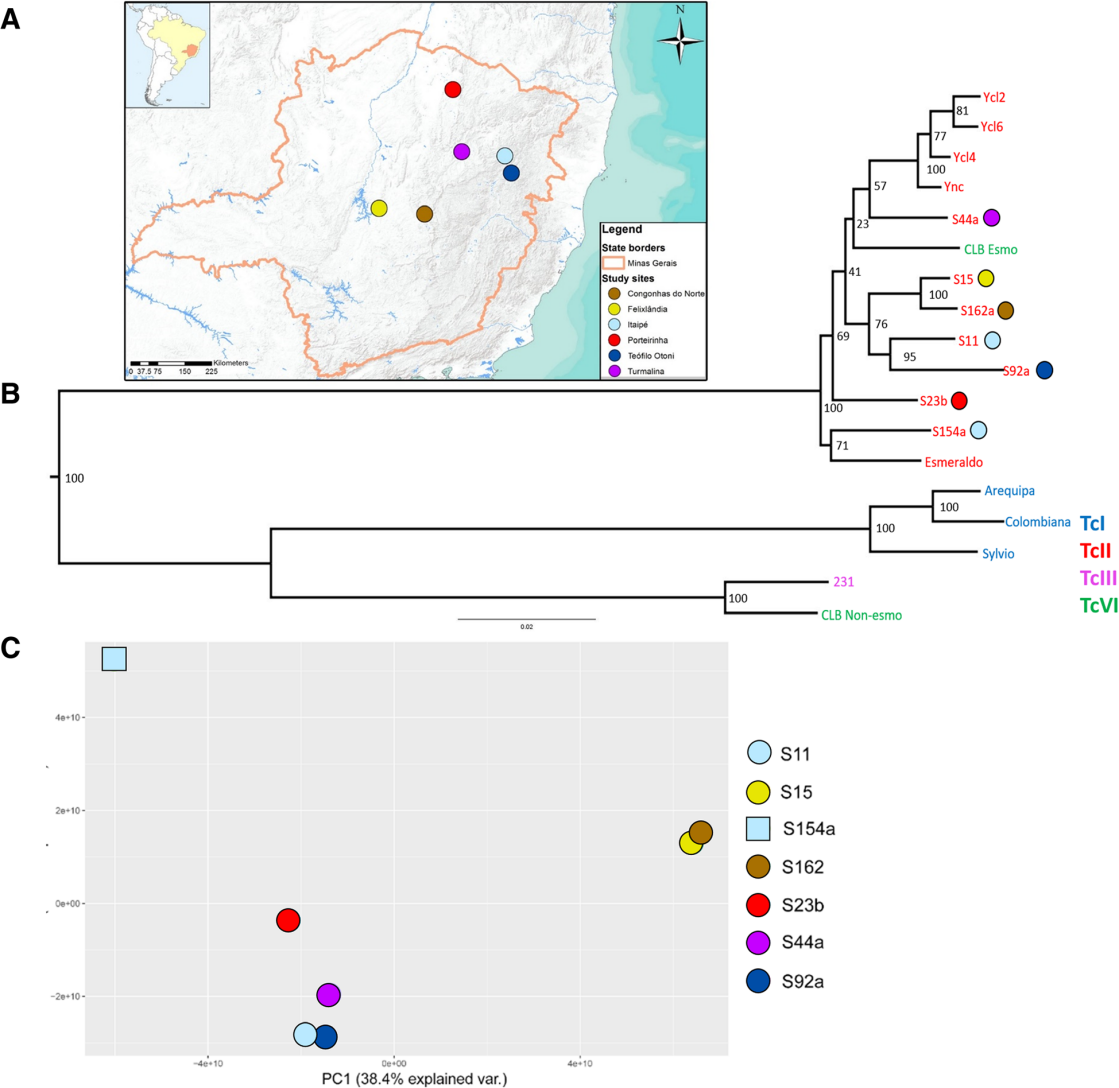
*T. cruzi* TcII field isolates: geographic distribution and phylogenomic analysis based on nuclear markers. **a** From the seven TcII samples evaluated, five were isolated from the northeast region of Minas Gerais: S11 (Itaipé - 17.3339^o^ S, 41.6411^o^ W), S154a (Itaipé - 17.3339^o^ S, 41.6411^o^ W), S23b (Porteirinha - 15.7032^o^ S, 43.0583^o^ W), S44a (Turmalina − 17.2856^o^ S, 42.7269^o^), and S92a (Teófilo Otoni - 17.8600^o^ S, 41.5091^o^ W); while two were obtained from the central region of Minas Gerais: S15 (Felixlândia - 18.7136^o^ S, 44.9253^o^ W) and S162a (Congonhas do Norte - 18.8242^o^ S, 43.6759^o^ W). **b** Maximum likelihood phylogenomic analysis of the TcII field isolates and previously sequenced *T. cruzi* from different DTUs, based on 794 nuclear single copy conserved genes. The colored circles correspond to the geographic localization in which each TcII isolate was obtained. The numbers correspond to the percentage of bootstrap replicates that supported each branch, where 100% corresponds to the 1,000 replicates **c** PCA plot based on the SNPs present in the seven TcII field isolates. Ync = Y strain non-cloned population; CLB-Esmo = CL Brener Esmeraldo-like haplotype; CLB-Nonesmo = CL Brener Non-esmeraldo-like haplotype

To evaluate the genomic diversity among the *T. cruzi* TcII DTU within close geographic regions, we sequenced, de novo assembled and compared the nuclear and mitochondrial genome sequences of seven TcII field isolates. The assembly statistics for the nuclear genomes are available in the Additional file 1: Table S1. These parasites were isolated from chagasic patients from the central (S15 and S162a) and northeastern (S11, S23b, S44a, S92a, S154a,) regions of the Minas Gerais state, Brazil (Fig. 1a).

### *T. cruzi* nuclear and mitochondrial phylogeny

From a total of 1,563 nuclear single copy genes that are conserved among the CL Brener Esmeraldo and Non-Esmeraldo-like haplotypes [28], 794 were partially de novo assembled in all seven TcII field isolates (Additional file 2: Table S2), while the other 769 genes were absent in at least one of the assemblies. From these 794, 701 genes were partially recovered after the Gblocks analysis (Additional file 3: Table S3), totalizing 558,587 nucleotides, which were used to estimate the nuclear maximum likelihood phylogeny of these strains. To better classify the TcII field samples, other strains from the DTUs TcI (Arequipa, Colombiana and Sylvio), TcII (Y strain and clones - Ycl2, Ycl4, Ycl6 - and Esmeraldo), TcIII (231), and TcVI (CL Brener Esmeraldo-like haplotype and CL Brener Non-Esmeraldo-like haplotype) were also included in this analysis (Fig. 1b). All the evaluated TcII strains/isolates clustered together and separated from the TcI and TcIII strains. As expected, the CL Brener (TcVI) Esmeraldo-like haplotype, which is derived from a TcII ancestor, clustered together with TcII strains. Similarly, the CL Brener (TcVI) Non-Esmeraldo-like haplotype, derived from a TcIII ancestor, clustered with the 231 (TcIII) strain. Concerning the TcII field isolates, two pair of samples, S15-S162a and S11-S92a, which were isolated from close geographic regions, were also clustered in the phylogenetic analysis, suggesting that for these isolates, genomic diversity correlates with geographic distances. On the other hand, the strains S11 and S154a that were isolated from the same locality had a distant phylogenetic relationship, suggesting that although geographic distance may correlate with TcII genomic diversity, there are different strains simultaneously coexisting in the same area (Fig. 1b). A principal component analysis (PCA) of all the differential nuclear SNPs found in the seven TcII field isolates also clustered together S15 - S162a and S11 - S92a and indicated S154a as a distantly-related isolate among the studied field samples (Fig. 1c).

**Fig. 2:**
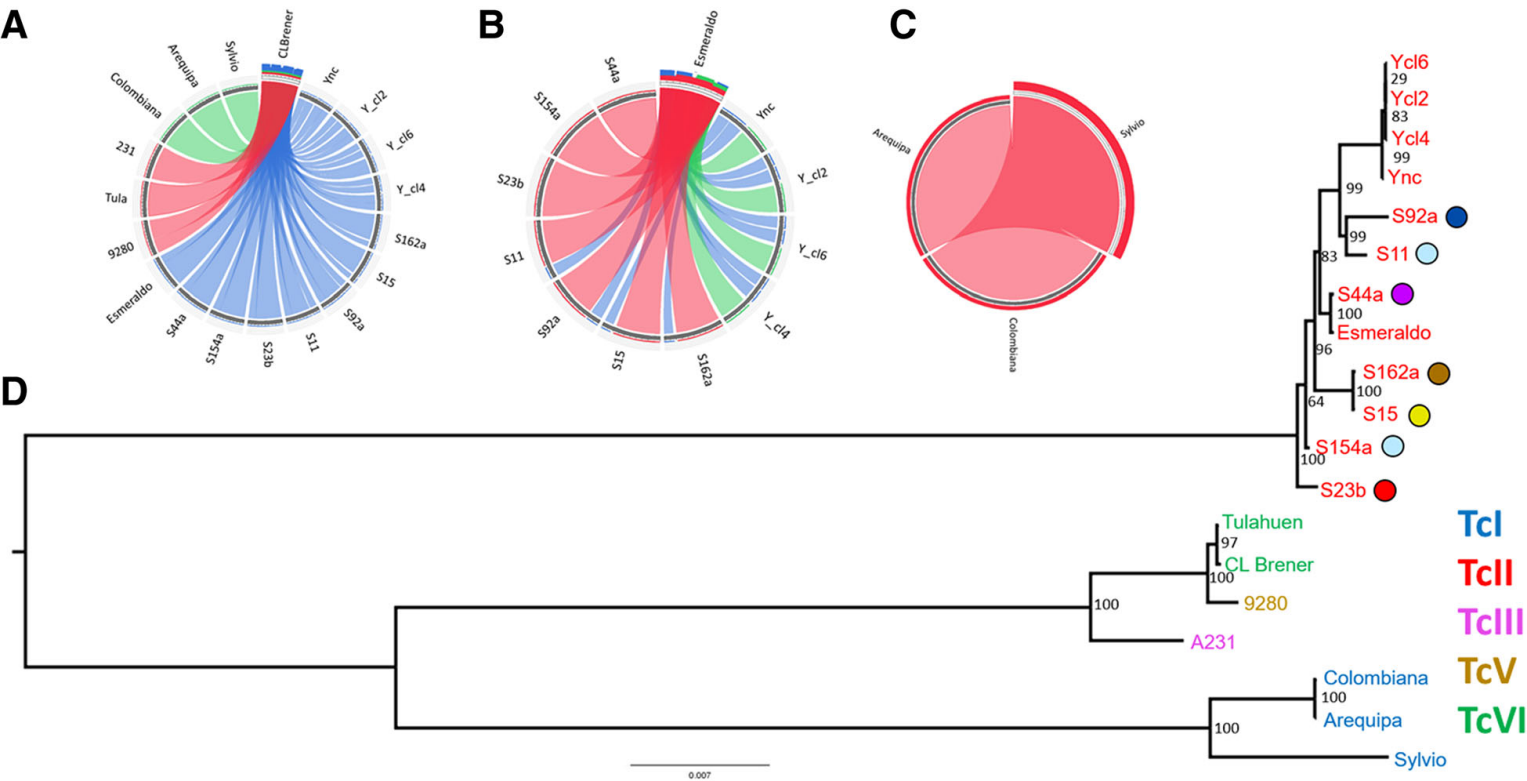
*T. cruzi* maxicircle phylogenetic analysis. Circos plot of the similarity along the assembled maxicircle sequence between: **a** *T. cruzi* strains from different DTUs using the CL Brener maxicircle sequence as reference; **b** Only TcII strains, using Esmeraldo maxicircle as reference; **c** Only TcI strains, using Colombiana maxicircle as reference. The colors represented in the circos plot are ranked from red to blue and are related to the best hits based on their BLAST bit-scores. **d** Maximum likelihood phylogenetic analysis of the maxicircle sequence among the TcII field isolates and *T. cruzi* maxicircles from different DTUs. The colored circles correspond to the geographic localization in which each TcII sample was obtained as represented in Fig. 1a). The numbers correspond to the percentage of bootstrap replicates that supported each branch, where 100% corresponds to the 1,000 replicates Ync = Y strain non-cloned population

Comparison of the gene conservation among the 19 newly assembled maxicircles, showed substantial differences in the kDNA sequences among *T. cruzi* DTUs (Fig. 2a). The CL Brener maxicircle shares higher identity (bit score > 40,000) with sequences from Tulahuen, 231 and 9280; an intermediate identity (bit score > 19,000 < 40,000) with TcI strains (Arequipa, Colombiana and Sylvio) and a lower identity (bit score > 4,000 < 19,000) to TcII strains (S15, S23b, S44a, S154a, S162a, S11, S92a, Esmeraldo, Y clones and Y population) (Fig. 2a). Next, we compared the variability within TcII DTU kDNAs, using as root the Esmeraldo maxicircle (Fig. 2b). This analysis showed differences among TcII maxicircle sequences, where Esmeraldo sequence was closely related to the TcII field isolates, specially S44a, S154a and S23b (bit score > 37,000), and less similar to the Y clones and population (bit score > 18.000 < 37,000). On the other hand, when only TcI maxicircles were evaluated, we found that they shared equally most of their sequences (Fig. 2c). Phylogenetic analysis of the 19 *T. cruzi* strains based on maxicircle coding genes separate the DTUs in a similar way to what was found with the nuclear phylogeny, with well-defined TcI and TcII clusters. Also, maxicircle analysis clustered TcV and TcVI strains closer to TcIII, reinforcing that the mitochondria from the hybrids DTUs TcV and TcVI were originated from the TcIII ancestor (Fig. 2d). As seen in the nuclear phylogeny, the two pairs of TcII strains from close geographic regions, S15-S162a and S11-S92a, also clustered in the maxicircle-derived phylogeny, while the strains from the same locality, S11 and S154a, had a distant phylogenetic relationship.

**Fig. 3:**
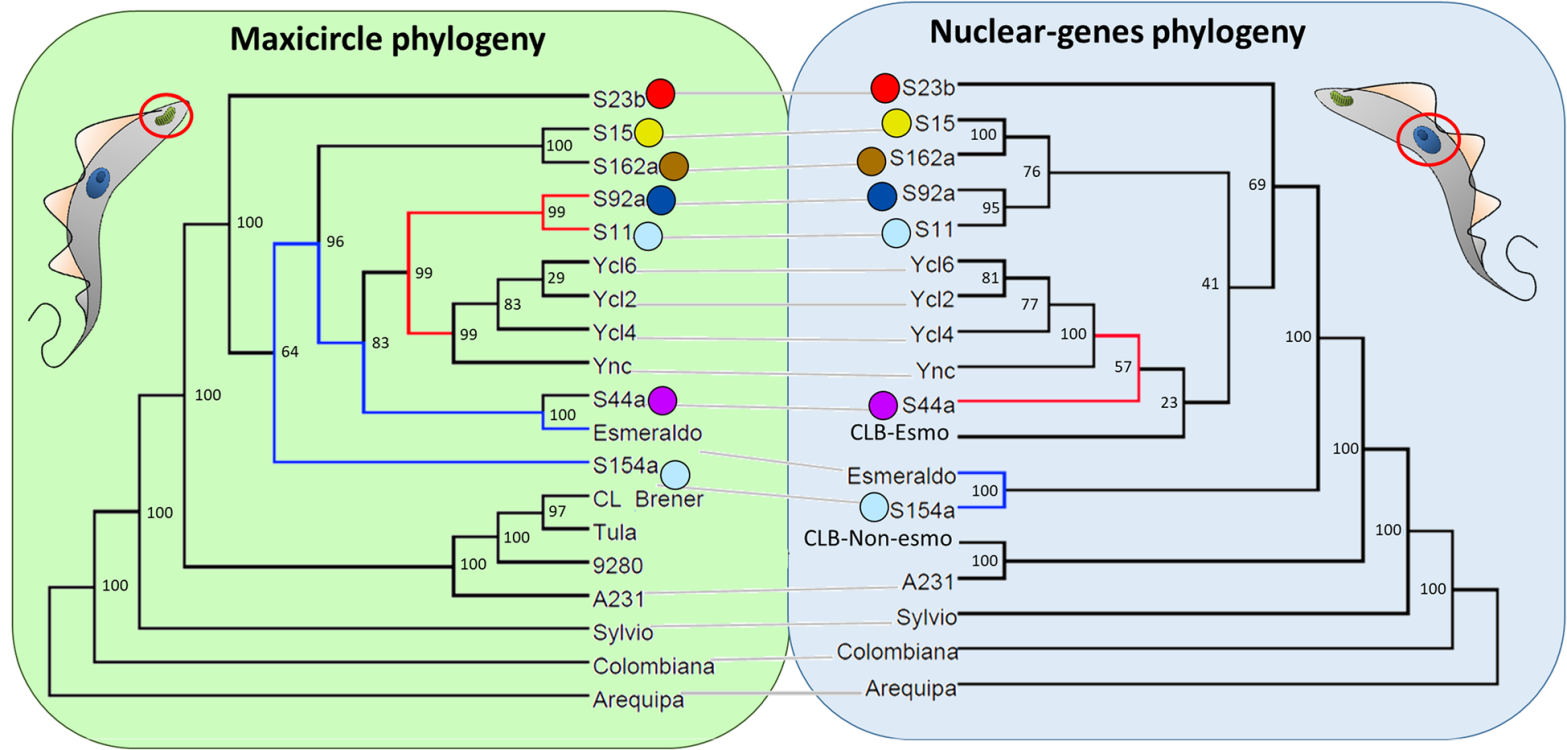
*T. cruzi* nuclear and maxicircle phylogenetic tanglegram. Comparison between the phylogeny based on nuclear single copy genes and the phylogeny based on the maxicircle sequences. Both trees were rooted by the Arequipa strain. The tree branches were reorganized (but not re-ordered) to facilitate the linking of the two trees. The sister group of the Y strain is highlighted in red, while the distance between Esmeraldo and S154a is highlighted in blue in both trees. The colored circle represents the geographic distribution of each sample, as depicted in Fig. 1a. The numbers correspond to the percentage of bootstrap replicates that supported each branch, where 100% corresponds to the 1,000 replicates Ync = Y strain non-cloned population; Tula = Tulahuen; CLB-Esmo = CL Brener Esmeraldo-like haplotype; CLB-Non-Esmo = CL Brener Non-esmo like haplotype

A comparative tanglegram between the nuclear and mitochondrial *T. cruzi* phylogeny showed large correspondences in most of the branches between both methodologies (Fig. 3), however, some discordances were also observed. Based on the maxicircle phylogeny, the sister group of the Y strain is the S11/S92a clade, while in the nuclear phylogeny, the Y sister group was S44a. The Esmeraldo sequence clustered with S154a in the nuclear based phylogeny, while clustered with S44a, but not with S154a, in the mitochondrial phylogeny. Finally, the four TcII isolates S15, S162a, S11 and S92a clustered together in the nuclear phylogeny but not in the maxicircle phylogeny. These discordances between nuclear and mitochondrial phylogeny could be caused by mitochondrial introgression events, where a parasite could inherit the mitochondria and nuclear genomes from different strains, resulting in discordant nuclear and mitochondrial phylogenies.

To search for evidences of mitochondrial heteroplasmy within TcII field isolates, we re-mapped the kDNA reads from each *T. cruzi* TcII sample in its reference-based assembled maxicircle sequence, and searched for heterozygous SNPs positions (Additional file 4: Figure S1). A total of 38, 46, 33, 29, 40,27, 36, 16, 17 and 21 heterozygous SNPS were found, respectively, in the strains S11, S154a, S15, S162a, S23b, S44a, S92a, Ycl2, Ycl4, Ycl6 (Additional file 4: Figure S1A). The majority of these SNPs were localized in non-coding or repetitive regions (Additional file 4: Figure S1 B and C).

### Chromosomal copy number variation

**Fig. 4:**
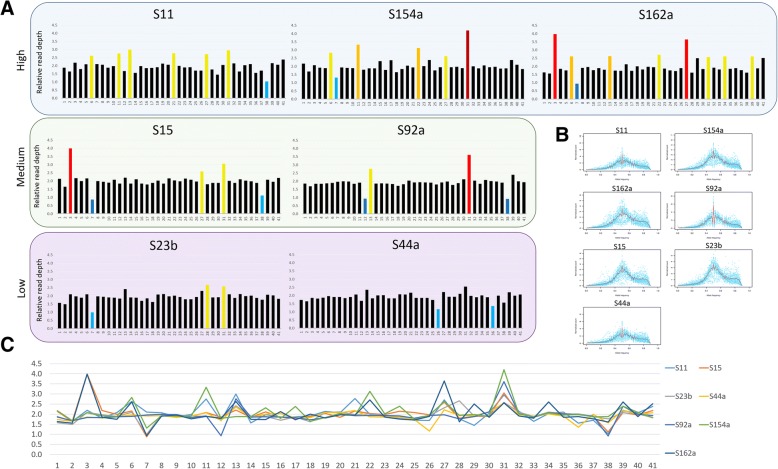
Predicted ploidy of *T. cruzi* field isolates**. a** The predicted ploidy of each chromosome from the *T. cruzi* field isolates S11, S154a, S162a, S15, S92, S23b, S44a, using as a reference the 41 CL Brener chromosome sequences, was estimated based on the median coverage of all *T. cruzi* genes, excluding those belonging to the largest multigene families. Each bar corresponds to the ratio between the genes median RDC in the chromosome and the genome coverage, representing its predicted chromosome copy number. Dark-blue, light blue, black, yellow, orange, red and brown bars correspond, respectively, to chromosomes with statistically supported predicted somies of 1; 1.5; 2; 2.5; 3; 3.5; 4. **b** The overall genomic ploidy of each strain was estimated by allele frequency of heterozygous SNPs, where a tendency of 0.4–0.5 represents a predominantly diploid genome. **c** Comparison of the predicted ploidy of each chromosome among TcII strains. Each line corresponds to a TcII strain

Analysis of chromosome copy number variation (CCNV) revealed large differences in the chromosomal duplication/loss events, highlighting the extensive ploidy variability found in this DTU (Fig. 4). The isolates S11, S154a and S162a presented more than 5 chromosomal duplication/losses, while S15 and S92 presented 5 and 4, and S23b and S44a had 3 or less aneuploidies with statistical significance (Fig. 4a, Additional file 5: Figure S2 and Additional file 6: Table S4). Interestingly, the phylogenetically close S15 and S162a isolates presented a similar chromosomal duplication/loss pattern, especially in the chromosomes 3, 7, 27 and 31 but a variable pattern in others, such as the chromosomes 6, 13, 22, 34, 38 and 39, suggesting that the duplication/loss of chromosomes is an ongoing process in *T. cruzi* evolution (Fig. 4c). Similar results were obtained for the closely related S11 and S92 strains, which share expansions in the chromosomes 13 and 31, but not in the 6, 11, 21 and 27 chromosomes. To determine the overall genomic ploidy of each *T. cruzi* TcII field isolate, the allele frequency of heterozygous SNP in the whole genomes was estimated. The allele frequency distribution peak of 0.5 for all the strains reinforces the predominance of diploid chromosomes in *T. cruzi* (Fig. 4b).

**Fig. 5:**
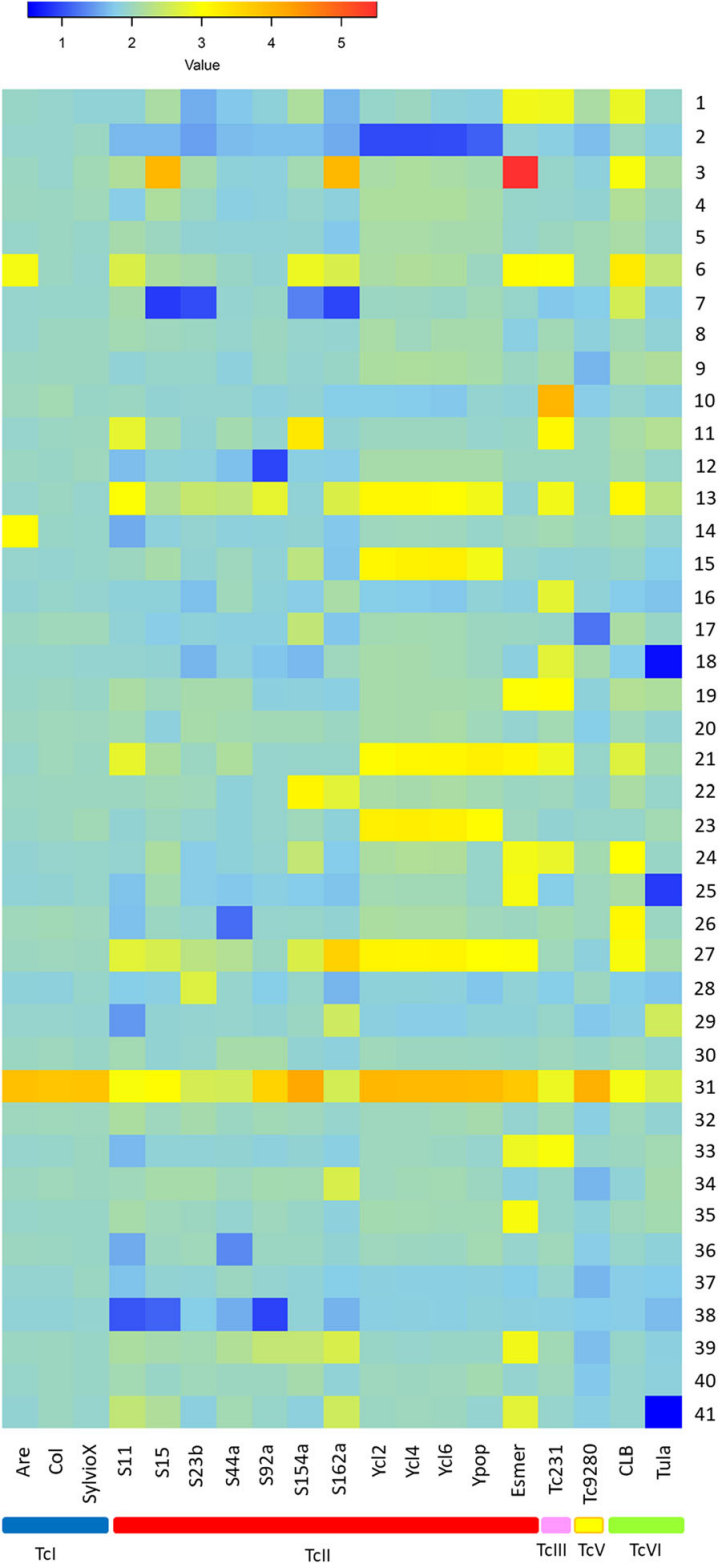
Chromosomal copy number variation among the *T. cruzi* DTUs and field isolates. The CCNV for each *T. cruzi* strain was performed based on the median coverage of all *T. cruzi* genes, excluding those belonging to the largest multigene families, normalized by the genome coverage. In this heatmap, a dark-blue, light blue, yellow, orange and red boxes correspond to, respectively, ~ 1; ~ 2; ~ 3; ~ 4 and ~ 5 chromosomal copies. Each line corresponds to one *T. cruzi* chromosome, numbered from 1 to 41, while each column represents a *T. cruzi* strain/isolate, in this order: TcI: Arequipa, Colombiana, Sylvio; TcII: S11, S15, S23b, S44a, S92a, S154a, S162a, Ycl2, Ycl4,Ycl6, Ypop, Esmeraldo; TcIII: 231; TcV: 9280; TcVI: CL Brener, Tulahuen

To compare the CCNV pattern of the TcII field isolates with other DTUs, the chromosomal duplication/loss pattern in all 19 *T. cruzi* strains evaluated by this work was estimated, showing several distinct patterns among and within DTUs, with some chromosomes being consistently duplicated or deleted (Fig. 5). The chromosome 31 was supernumerary in most of the strains from the five evaluated DTUs, while the chromosomes 6, 13, 27 also had an overall tendency to polyploidy, but in a lower extent than chromosome 31. There is also evidence for loss of chromosomes, as chromosomes 2, 7 and 38, which were in a haploid state in several *T. cruzi* strains. Next, to evaluate if the CCNV also vary within a given parasite population, we compared the chromosomal duplication/loss pattern in Y strain and three clones derived from this strain: Ycl2, Ycl4 and Ycl6. All the Y clones had a similar pattern of CCNV with each other and with the Y strain (non-cloned population), indicating that the CCNV pattern may be constant within a population (Fig. 5). Interestingly, the Y strain chromosome 11 had a drastic change of RDC starting at the 248-kb position, as seen in Reis-Cunha 2015 [28], resulting in an initial haploid and a terminal diploid state. To investigate if this difference is a result of mosaic aneuploidy in the population, where some cells have a complete chromosome 11 and others have a deletion of the initial 248 kb, or if within individual cells one homolog chromosome have a complete and the other one a truncated copy, we compared RDC along the entire chromosome in the Y strain and clones. The initial 248 kb of the three Y clones presented half of the RDC from the remaining chromosomal sequence, in a similar way to what was found in the Y strain (Additional file 7: Figure S3). This suggests that in individual parasites of Y strain, one copy of the CL Brener chromosome 11 is complete and the other one has an arm loss, resulting in a haploid state in its initial 248 kb sequence. Another possibility is that in Y strain, the corresponding CL Brener chromosome 11 is divided in two smaller chromosomes, a monosomic chromosome that correspond to the initial 248 kb and a disomic chromosome that corresponds to the remaining CL Brener chromosome 11 sequence.

**Fig. 6:**
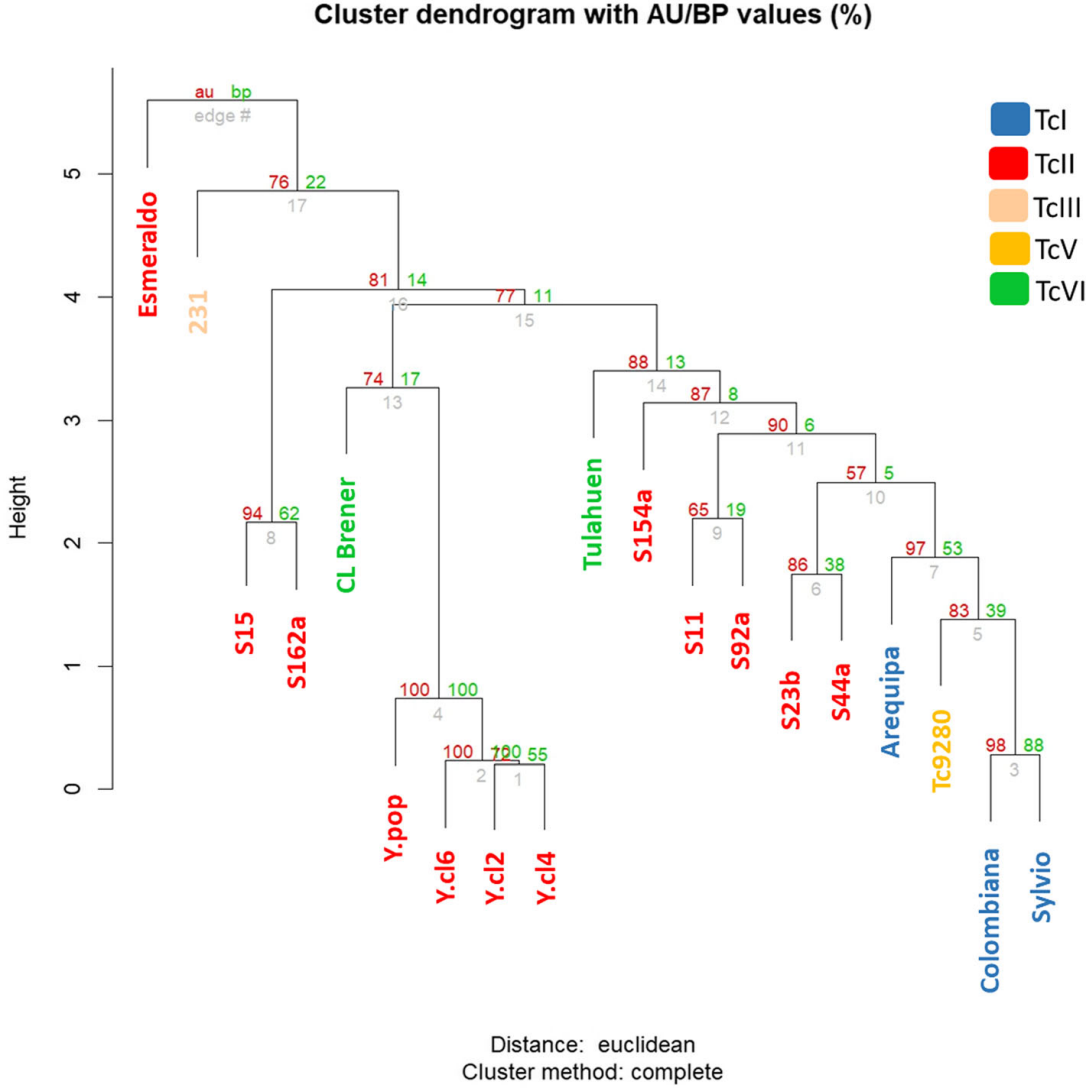
Dendrogram of the hierarchical analysis based on *T. cruzi* chromosomal copy number variation patterns. The hierarchical clustering analysis based on Euclidean distances of the predicted ploidy of each chromosome of the 19 *T. cruzi* samples was performed using the R package Pvclust. Two bootstrap resampling methods were employed to assess the uncertainty in the hierarchical cluster analysis: approximately unbiased (au) in red and bootstrap probability (bp) in green

A hierarchical clustering analysis based on the Euclidian distances of the predicted ploidy of each chromosome in the 19 *T. cruzi* strains showed that the CCNV events do not follow the parasite phylogeny, as TcI, TcV and TcVI strains were clustered within TcII strains (Fig. 6). Interestingly, the Esmeraldo (TcII) and 231 (TcIII) strains presented the most divergent CCNV pattern among the evaluated strains/isolates. Sequencing of additional TcIII isolates are required to evaluate the extent of ploidy variation within this DTU. The TcII field isolates that were clustered together in the nuclear and mitochondrial phylogeny, S15-S162a and S11-S92a, also clustered together based on the CCNV profile.

## Discussion

*T. cruzi* population genetics analysis are usually based on multilocus sequence typing of nuclear or mitochondrial markers, and therefore the comparison of the parasite variability is restricted to a number of genomic regions [10, 21, 26, 43–45]. On the other hand, the use of next generation sequencing (NGS) reads provides a wide genomic characterization of the parasite variability, allowing not only a comparison based on a broader set of genes, but also the correlation of chromosomal amplification/loss patterns with the parasite phylogeny. Most of the *T. cruzi* molecular studies are focused on the TcI DTU, the oldest and most widespread genetic lineage of the parasite, which is responsible for the majority of human infections from the Central America to Bolivia [5, 24, 26, 44–46]. There are, however, fewer studies concerning the variability, distribution and intercrossing of TcII, one of the most relevant *T. cruzi* subgroups related to human infection in the South America southern cone [5, 21, 47, 48]. Although the correspondence between DTU and clinical course is still unclear, some evidences point toward TcII association with severe manifestations of Chagas disease, presenting both cardiac and digestive manifestations, highlighting the importance of TcII for Chagas disease clinical outcomes [5, 24, 49]. To further explore the TcII variability, we sequenced the whole nuclear and mitochondrial genome of seven TcII isolated from patients from different locations in the Minas Gerais state, Brazil (Fig. 1a), and compared these isolates with each other and with reference strains from different DTUs. This is the first study to evaluate genome wide variation in *T. cruzi* isolates from close geographic locations, based on NGS.

### Nuclear and mitochondrial phylogeny

The first step to estimate correlations among evolution, chromosomal duplication/loss and recombination within isolates from *T. cruzi* TcII DTU was the assessment of their phylogeny based on a set of nuclear conserved single copy genes as well as based on all mitochondrial genes [28]. Single copy genes are ideal molecular markers to infer phylogeny due to their uniqueness and conservation across and within species groups. Especially in analysis based on short Illumina reads, as de novo assembly or mapping of these small reads to repetitive regions (as microsatellites) could result in artefactual variations. However, the use of single copy genes prevents mapping errors and false SNPs that could compromise phylogenetic conclusions. Even though single-copy genes do not diverge as often as microsatellites, the use of a large dataset provided enough resolution to separate TcII from other DTUs and allowed inferences of phylogenetic proximity among these field samples (Fig. 1b). The simultaneous evaluation of nuclear and mitochondrial markers allows a thorough evaluation of a lineage evolutionary history, due to the different inheritance patterns and mutation rates presented by these two genomic sequences. *T. cruzi* hybrid strains present uniparental inheritance of its mitochondrial DNA and bi-parental inheritance of its nuclear genome, as seen in CL Brener (TcVI) where the mitochondria was originated from the TcIII ancestor and the nuclear genome is composed by sequences derived from TcII and TcIII ancestors [50, 51]. Besides, events of mitochondrial introgression and heteroplasmy have already been described in *T. cruzi* [26], which could be a consequence of intra or inter-DTU-hybridizations events.

A comparison of the phylogeny based on the nuclear single copy genes and the mitochondrial genes revealed that most of the branches are shared by the maximum likelihood phylogeny estimations by markers from both sequences (Fig. 3). The two samples from the central region of Minas Gerais, S15 and S162a, clustered together with high bootstrap value (100%) in both nuclear and mitochondrial phylogenetic analysis as well as in the PCA plot based on whole genome SNPs (Figs. 1b, c and 2d). These two samples are separated from the other five TcII field isolates by the Espinhaço Mountain, a mountain range extending from the central region of Minas Gerais to the northern region of the Bahia State, Brazil. This geographic barrier could restrict the transit of insect vectors, separating *T. cruzi* populations from the central and north/northeastern regions of the state. The S154a sample was the outgroup of all TcII field samples based on the single copy genes phylogeny, clustering together with Esmeraldo (Fig. 1b) and presented the most divergent SNP pattern based on PCA analysis of whole nuclear genomic SNPs from the seven TcII field isolates (Fig. 1c). This suggests that S154a lineage could have endured several recombination events, being the most mosaic sample from the TcII isolates evaluated, or that it have diverged early from the other TcII field isolates. To date, the majority of field evidence supports that *T. cruzi* is not strictly clonal, and that recombination is a nonobligatory yet common event [16, 17, 21, 23, 26, 44]. The occurrence of recombination among *T. cruzi* populations was documented in TcI from Bolivia [45, 46], Colombia [22] and Brazil [44], as well as among TcII strains from Brazil [21], based on samples from close geographic regions. *T. cruzi* strains were also capable of genetic recombination in laboratory, presenting fusion of parental genotypes, loss of alleles, homologous recombination and uniparental inheritance of kinetoplastid maxicircle genome [30]. Although *T. cruzi*-related parasites *Leishmania* sp. and *T. brucei* appears to undergo meiotic events in the insect vector [52–54], *T. cruzi* genetic exchange appears to occur in the mammalian host and is independent of an meiotic stage [16]. However, the rate in which these events occur in *T. cruzi* and whether recombination may also occur within insect vector is still unknown.

There were some divergences among the phylogeny of TcII field isolates, based on nuclear or mitochondrial genes (Fig. 3). The sister group of the Y clones/strain was S44a based on the nuclear single copy genes, and S11/S92a based on the mitochondrial genes (Fig. 3 - Red line). However, the bootstrap values supporting this nuclear branching was low. Similarly, Esmeraldo strain clustered with S154a based on nuclear markers and with S44a based on the maxicircle phylogeny (Fig. 3 – Blue line). This difference in the nuclear and maxicircle phylogeny are probably caused by mitochondrial introgression events, as recombination and gene exchange appears to be a common event in *T. cruzi* [20, 44]. Mitochondrial introgression was already been documented in some *T. cruzi* strains isolated from North and South America [10, 11, 25, 26]. Although the biological implications of mitochondrial introgression are still unknown, its occurrence reinforces the reliability of recombination inferences among *T. cruzi* strains. To search for mitochondrial heteroplasmy, the presence of heterogeneous mitochondrial genomes in an individual cell, we re-mapped the mitochondrial reads of each TcII strain to its reference-based maxicircle assembly and looked for heterozygous SNPs (Additional file 4: Figure S1). Only a few number of heterozygous SNPs were identified in the mitochondrial genome of TcII strains/isolates, and most of them were localized in repetitive regions, not supporting the occurrence of mitochondrial heteroplasmy. To date, reported levels of mitochondrial heteroplasmy in *T. cruzi* are scarce [26]. Heteroplasmy was already observed in the Sylvio X10/1 (TcI), based on re-mapping of its kDNA reads to the reference Sylvio X10/1 maxicircle, resulting in a total of 74 SNPs in eight genes and three intergenic regions [26]. In our analysis, we found only 1–3 heterozygous SNPs in coding genes, not providing enough support for heteroplasmy inferences. However, the absence strong evidence of heteroplasmy in our samples could be an underestimation resulting from low coverage (~60X), when compared to 163x coverage used in the Sylvio X10/1 study [26], as only 12.2% of the reads corresponded to the minor variant SNPs in Sylvio [26].

### Chromosomal copy number variation

Chromosomal copy number variation appears to be well tolerated in the trypanosomatids *T. cruzi* [27, 28] and *Leishmania* [55–61]. In the present work, we compared the CCNV pattern of seven TcII field samples with each other (Fig. 4), as well as with previously sequenced strains from the TcI, TcIII, TcV and TcVI DTUs (Fig. 5). It is well known that *T. cruzi* strains have distinct profiles of chromosomal bands based on Pulse Field Gel Electrophoresis analysis, and therefore a variable karyotype among DTUs. These differences where mainly attributed to expansion/retraction of multigene families clusters, or to events of chromosomal fusion/break during *T. cruzi* evolution [34, 51, 62–65]. Despite these variations, the housekeeping genes clusters are highly conserved and syntenic among the parasites strains and therefore represent an adequate source for sequence normalization in CCNV analysis [27, 28, 34, 66–68]. A hierarchical cluster dendrogram based on the CCNV pattern of the 19 *T. cruzi* strains from different DTUs grouped TcI, TcV and TcVI samples within TcII clusters, showing that the chromosomal duplication/loss events do not follow the phylogeny based on nuclear single copy genes or mitochondrial markers (Fig. 6). In fact, all the TcII strains evaluated had a different pattern of CCNV, with low (S23b, S44a) medium (S15, S92a) or high (S11, S154a, S162a) number of aneuploidies (Fig. 4), in accordance with which was previously observed between the TcII strains Esmeraldo and Y [28]. This suggests that chromosomal gain/loss is frequent in *T. cruzi*, and occurs in a higher rate than DTU branching events, varying among and within DTUs [27, 28]. The aneuploidy pattern also varies within close geographic populations of *Leishmania donovani* [55], reinforcing that both parasites are naturally aneuploids [69]. Based on FISH analysis, CCNV was identified within the same population in several *Leishmania* species and strains [57, 58, 70]. To explain this observation, a model based on miss segregation or stochastic replication of chromosomes was proposed in *Leishmania* [57, 58, 70]. In this model, there is an asymmetric replication and allotment of chromosomes during mitosis, resulting in polyploid and haploid cells. For this reason, the *Leishmania* population present a “mosaic aneuploidy”, where cells from the same population presented different patterns of aneuploidies, and the most prevalent genotype within a population was estimated as ~ 10% of the cell counts [57, 58]. To evaluate if the aneuploidy pattern in *T. cruzi* also varies in a similar rate, we have cloned and sequenced the whole genome of three clones derived from Y strain, based on RDC. All the three Y clones as well as the Y strain [28] presented a similar aneuploidy pattern (Fig. 5), suggesting that although CCNV in *T. cruzi* varies among and within DTUs, it seems constant within a given population, different from what is observed in *Leishmania*. This data is in accordance with pulse field gel electrophoresis assays denoting that the chromosomal bands from the D11 clone from the G strain of *T. cruzi* was stable in continuous culture isolates over several years [34, 71]. Similarly, the *L. donovani* strain BPK282/0 cl4 presented a stable aneuploidy pattern for at least 32 passages after genome sequencing [55]. However, this unaltered pattern of aneuploidies could be a consequence of the normalized sum of the CCNV from the entire population, precluding the identification of aneuploidy patterns from single cells. In fact, several *T. cruzi* strains had estimated chromosomal ploidies with intermediated values (as between 2 and 3), which could be a consequence of a mixed cell population with disomic and trisomic chromosomes [28, 72]. A process that could explain the generation of polyploid cells in *T. cruzi* is the fusion of two parental diploid or polyploid cells followed by a progressive reduction of the chromosome number, in a similar way as the parasexual cycle of *Candida albicans* [17, 29, 73]. In this model, the fusion of ‘parental’ cells is followed by karyogamy and reductional mitotic division, which would lead to aneuploid daughter cells with different genomic/genetic contents [17, 58]. FACs analysis of *T. cruzi* hybrid isolates revealed an increase of ~ 70% in their DNA content when compared to parental strains [33]. The subsequent prolonged maintenance of experimental hybrids in axenic cultures lead to a gradual and progressive reduction in DNA content [17, 33], further supporting the parasexual model as basis to the generation of aneuploidies in *T. cruzi*.

Although structural variability and aneuploidies are usually associated with detrimental phenotypes in complex eukaryotes [74–76], some unicellular eukaryotes rely on aneuploidy as a mechanisms to allow rapid adaptation to changing environments, suggesting that the variation in chromosome number could also have a positive fitness effect in stress conditions [73, 77, 78]. Aneuploidy it is a common feature in trypanosomatids, described in several *T. cruzi* strains and *Leishmania* species [28, 55, 56, 79], however it appears to be absent in *T. brucei* [80]. As *T. cruzi* and *Leishmania* have their genome divided in a large number of fragments (~ 34 to 47 putative chromosomes) [50, 51, 67, 81, 82], altering the copy number of specific chromosomes would alter the dosage of a restrict set of genes, avoiding detrimental consequences of large-scale dosage alterations. On the other hand, the diploid parasite *T. brucei* has its genome divided in eleven megabase-sized chromosomes [67, 81], suggesting that aneuploidies would be better supported in organisms that have its genome dispersed in a large number of chromosomes. However, the evaluation of a CCNV in a broader set of unicellular eukaryote species is necessary to confirm this hypothesis. Copy number variation is a well-documented mechanism to alter gene expression and enhance variability, especially in parasites that mostly regulate its gene expression post-transcriptionally as trypanosomatids [83–85]. Miss-segregation of chromosomes or the parasexual cycle could alter the copy number of several genes within a few generations, which may enable heteroxeneous parasites to rapidly adapt to the transition between the mammalian and invertebrate hosts [17, 60, 61, 72]. This hypothesis have been recently confirmed in *Leishmania*, where a shift in the pattern of duplicated/loss chromosomes was described as the parasite change from culture cells to insect vectors and to the mammalian host [61]. This shift in chromosome duplication patterns also impacted RNA levels, showing a higher expression of genes derived from polysomic chromosomes [61]. Alternatively, if a polysomic state is stable for long evolutionary periods it could allow the accumulation of mutations and consequent evolution of new functions for the duplicated genes, as the ancestral copy would still be present in the genome [72]. The gain or loss of a whole chromosome was already associated with increased fitness in stress conditions and drug resistance in *Saccharomyces cerevisiae*, *Candida albicans* and carcinomatous lung cancer cells [77–79, 86], and could also be explored by the parasites to allow natural selection of favorable phenotypes. In fact, CCNV was also associated with drug resistance in *L. major* and *L. infantum* based on transcriptional profiling using microarrays, southern blot and comparative genomic hybridization, where these chromosomes reverted to disomy in the absence of drug pressure [79, 87]. However, Downing 2011, based on RDC analysis found no clear link between aneuploidy and drug resistance in *L. donovani* clinical isolates [55]. Drug selection also appears to promote gene amplification and translocation in *T. cruzi* [88], showing that genomic expansion is a widespread process employed by trypanosomatid parasites to survive to environmental changes.

The chromosome 31 was the only one supernumerary in the majority of the *T. cruzi* evaluated strains, been consistently polyploid among isolates from different DTUs (Fig. 5), as previously seen in a more restricted number of strains [28]. This chromosome is enriched with genes related to glycoprotein biosynthesis and glycosylation processes, especially with genes related to mucin glycosylation and biosynthesis, as the enzyme UDP-GlcNAc-dependent glycosyl-transferase [28, 89]. Mucins are highly glycosylated proteins that covers the whole surface of the parasite, which are directly involved in its survival in both invertebrate and vertebrate hosts [89, 90]. One of the possible explanations for the expansion of chromosome 31 in *T. cruzi* could be the need to glycosylate the 2 × 10^6^ mucins that covers the parasite surface [28, 89, 90].

## Conclusions

Next generation reads from whole genome and mitochondrial sequencing allows the simultaneous evaluation of phylogeny, aneuploidy and allele frequencies in the same population of cells, providing a genome-wide evaluation of the variability among closely geographic field isolates [28, 55, 69]. Phylogenetic analysis of the TcII DTU suggested the occurrence of genomic recombination events during *T. cruzi* evolution in Minas Gerais, with possible mitochondrial introgression events. The discordance between the nuclear/mitochondrial phylogeny and the CCNV suggests that chromosomal gain/loss are more frequent than DTUs branching events in *T. cruzi*, and could be explored by the parasite to allow rapid selection of favorable phenotypes. Besides, the highly variable pattern of aneuploidies found within TcII field samples and the concordant pattern of CCNV within Y clones suggest that the parasexual cycle could be the major mechanism enrolled in genetic exchange and aneuploidy generation in geographically close *T. cruzi* isolates [17]. However, the miss segregation or stochastic replication of chromosomes, as proposed to *Leishmania* [57, 58], could also be a driving force in *T. cruzi* CCNV. To further address CCNV within a *T. cruzi* population, single-cell genome sequencing based analysis could provide a new level of resolution, comparing the whole chromosomal pattern of single parasites isolated from the same population. Aneuploidy constitutes a large source of adaptability, throughout gene dosage alterations and shaping of genetic heterogeneity [69], which could be important to the rapid adaptation and for the interchange between the invertebrate/mammal hosts in heteroxeneous parasites. Finally, the expansion of the chromosome 31 in a larger number of isolates/strains highlights the importance of the glycosylation to the *T. cruzi* survival.

## Methods

### Genome sequencing and read libraries processing

A total of 19 *T. cruzi* whole genome sequencing read libraries containing samples from TcI, TcII, TcIII, TcV and TcVI DTUs were used in this study. Eleven of these sequences were generated in this work using Illumina Hiseq2000 sequencer, with ~60x coverage, generating pair-end read libraries with 100 bp read size and insert size of 350 bp. They consisted of seven TcII strains recently isolated from the central (S15 and S162a) and northeastern (S11, S23b, S44a, S92a and S154a) regions of Minas Gerais state, Brazil; three clones from the Y strain (Ycl2, Ycl4, Ycl6); and one sample of the CL Brener (TcVI) strain. Other five *T. cruzi* whole genome and mitochondrial read libraries were generated by our group in a previous study [28] consisting of samples of TcI (Arequipa, Colombiana and Sylvio), TcII (Y strain) and TcIII (231). The remaining three samples were downloaded from the National Center for Biotechnology Information Sequence Read Archive (NCBI-SRA), consisting of samples from the TcII (Esmeraldo), TcV (9280) and TcVI (Tulahuen) DTUs. The detailed description of each read library is summarized in the Additional file 8: Table S5.

The quality of each read library was evaluated with the FASTQC tool (http://www.bioinformatics.babraham.ac.uk/projects/fastqc/) and filtered using Trimmomatic [91]. The phred filtering threshold was a minimum of 30 for Illumina reads and 20 for the 454 and Ion Torrent libraries, using a five nucleotide sliding window, as well as a minimum read size of 50 nucleotides.

The whole genome assembly contigs from all CL Brener Esmeraldo-like and Non-esmeraldo putative chromosomal sequences and unassigned contigs version 26 were downloaded from the TriTrypDB [92] (Additional file 9: Table S6).

### Parasite cloning and DNA isolation

For cloning the *T. cruzi* Y (TcII) strain, 10^3^ epimastigotes were plated into a semi-solid medium (low-melting agarose 0.75%, brain heart infusion 48.4%, liver infusion tryptose (LIT) 48.4%, 2.5% defibrinated blood, and 250 μg/mL penicillin/streptomycin) and incubated at 28 °C for 35 days. Single clones were obtained and transferred to 25 cm^3^ culture flasks with 5 mL of LIT medium and 10% fetal bovine serum. After cloning, the three Y clones (Ycl2, Ycl4 and Ycl6) epimastigote cultures where briefly cultured before DNA extraction. To isolate the parasite genomic and mitochondrial DNA, a total of 1 × 10^8^ Y epimastigotes were centrifuged at 3000 g for 10 min at 4 °C. The parasites where washed three times with ice-cold PBS, suspended in PBS with 300 μg/mL proteinase K and incubated at 25 °C for 10 min. The genomic DNA was obtained with the Wizard® Genomic DNA Purification Kit (Promega), following the manufacturer instructions. The extracted DNA was submitted to a genotyping protocol using three different previously described markers to confirm the DTU identity [11, 93, 94].

### Nuclear genome assembly

The genome assemblies of Esmeraldo, 231 and Sylvio strains, as well as the CL Brener Esmeraldo-like and Non-Esmeraldo haplotypes were downloaded, respectively, from the European Nucleotide Archive, NCBI and TriTrypDB (Links in the Additional file 9: Table S6). The genomes of the seven TcII field isolates, three Y clones, Y strain, Arequipa and Colombiana were de novo assembled, using Velvet optimizer with velvet version 1.2.10 [95, 96] for the Illumina, or using Celera 8.3 [97, 98] for the 454 read libraries. The NCBI accession numbers for the nuclear genome assemblies are listed in the Additional file 10: Table S7.

### kDNA assembly and sequence similarity visualization

To select the most suitable mitochondrial sequence to be used in reference-based maxicircle assemblies, the read libraries for each of the *T. cruzi* strains were competitively mapped to all three publically available maxicircle references using BWA-mem [99, 100]. The available mitochondrial genomes with their respective NCBI accession numbers were: TcI Sylvio (FJ203996.1), TcII Esmeraldo (DQ343646.1) and TcVI CL Brener (DQ343645.1). The reference with the highest coverage for each strain was selected as a template. Based on this analysis, Sylvio maxicircle was selected as reference for Arequipa and Colombiana strains, Esmeraldo maxicircle was selected as reference for all the TcII field isolates, as well as for Y strain and clones and the CL Brener maxicircle was selected as reference for 231, 9280 and Tulahuen strains (Additional file 11: Figure S4). The final FASTA consensus maxicircle genome sequence was generated by submitting the BAM files to a pipeline using SAMTools mpileup, disabling probabilistic realignment for the computation of base alignment quality, reducing the chance of false SNPs caused by misalignments (-B), bcftools view using the minimum allele count of sites and including all sites with one or more genotypes (−cg), vcfutils.pl to convert the bcftools vcf output file to a consensus fastq file (vcf2fq) and seqtk fq2fa to convert the fastq output to a final consensus fasta file [101]. The NCBI accession numbers for the maxicircle sequence assemblies obtained in this study are listed in the Additional file 10: Table S7. The maxicircle assemblies of the *T. cruzi* strains Sylvio (TcI), Esmeraldo (TcII) and CL Brener (TcVI) were downloaded from the aforementioned databases. To visualize the similarity patterns and differences between each one of the maxicircle sequences, a BLASTn search [102] between all samples with an e-value cutoff of 1e^− 20^ was performed and submitted to Circoletto [103], a Circos program package [104].

### Phylogenetic analysis

The nuclear phylogeny of 17 from the 19 *T. cruzi* samples was determined based on 1,563 CL Brener esmeraldo-like haplotype single copy nuclear genes described in Reis-Cunha 2015 [28]. These sequences were recovered from the assembled contigs of the aforementioned samples using BLAT [105], where only genes that were identified in all the assembled genomes where kept and used in the phylogenetic analysis. Tulahuen and 9280 strains were excluded from this analysis as their hybrid origin hampered the quality of the nuclear genome de novo assembly. For the kDNA phylogeny, all the 19 *T. cruzi* samples were used, including Tulahuen and 9280. For both nuclear and mitochondrial genomes, each one of the recovered genes were aligned using MUSCLE [106] and the poorly aligned or gaps regions were eliminated using Gblocks [107]. The best fitting nucleotide substitution model for the phylogenetic analysis was determined using Jmodeltest [108]. The maximum likelihood phylogenetic tree was built using the PhyML [109], with the Generalized Time Reversible (GTR) model 1,000 bootstrap replicates, 0.9 proportion of invariable sites, 0.93 gamma distribution for the nuclear and 0.27 gamma distribution for the mitochondrial genome. The final phylogenetic tree images were built using FigTree v.1.4.2 software (http://tree.bio.ed.ac.uk/software/figtree/). A comparative tanglegram based on the nuclear and mitochondrial markers were generated, using the program Dendroscope [110].

### Principal component analysis

To estimate the distance among the seven TcII field isolates based on whole genome differential SNPs, a consensus nuclear genomic sequence was generated to each sample, using the GATK FastaAlternateReferenceMaker (https://software.broadinstitute.org/gatk/documentation/tooldocs/current/org_broadinstitute_gatk_tools_walkers_fasta_FastaAlternateReferenceMaker.php). Then, a distance matrix based on differential SNPs was generated and loaded in the R caret package to generate the PCA plot (http://topepo.github.io/caret/index.html).

### Chromosomal copy number variation

The estimation of the copy number of each chromosome from each strain was based on the median coverage of all genes present in a given chromosome excluding those that belong to the largest *T. cruzi* multigene families (trans-sialidase, MASP, TcMUC, RHS, DGF-1 and GP63) and the ones that had an outlier coverage based on Grubb’s test. Briefly, *T. cruzi* CL Brener chromosomal reference sequences version 26 were downloaded from the TriTrypDB [92]. Then, the read libraries from the TcI and TcIII strains where mapped to the Non-Esmeraldo-like chromosomes, while strains from the TcII, TcV and TcVI were mapped to the Esmeraldo-like chromosomes [50] using BWA MEM [100]. The mapped reads were filtered by mapping quality 30 using SAMtools v1.1 [101], the RDC of each position in each chromosome was determined with BEDtools genomecov v2.16.2 [111] and *in-house* Perl scripts. For each chromosome, genes with outlier coverages were excluded, based on iterative Grubb’s test, with *p* < 0.05. The median RDC of all non-outlier genes in each chromosome was normalized by the genome coverage (estimated as the mean RDC of all single-copy genes in all chromosomes for each strain) and assumed as the chromosomal somy (Additional file 12: Figure S5A). Finally, the statistic support that a given chromosome somy was lower than 1; 1.5 or higher than 2; 2.5; 3; 3.5; 4; 4.5 or 5 was performed based on Mann-Whitney-Wilcoxon tests, with one-way analysis of variance and a significance of *p* < 0.05, using R. A list containing all the genes used to estimate each chromosome somy of all seven TcII field isolates can be seen in the Additional file 13: Table S8.

Single-nucleotide polymorphisms (SNPs) of the mapped reads from all the *T. cruzi* strains were obtained using SAMtools mpileup function [101]. To be considered as a reliable SNP, the position RDC must be at least 10. For each chromosome, the proportion of read depth in alleles in each predicted heterozygous site was obtained and rounded to the second decimal place. Base frequencies were rounded in one hundred categories, ranging from 0.01 to 1, and an approximate distribution of base frequencies for each chromosome was obtained by Perl scripts and plotted in R (www.r-project.org, R Development 2010) (Additional file 12: Figure S5B). To estimate the overall ploidy of each genome, the same methodology was applied, but the heterozygous positions from all CDSs from all chromosomes were employed simultaneously.

### Aneuploidy pattern dendrogram

A hierarchical clustering analysis based on the predicted CCNV in all *T. cruzi* strains was performed with the R implemented Pvclust package [112]. A distance matrix was built with pairwise Euclidean distances between the strains, and the dendrogram was generated by complete linkage method. To assess the uncertainty in hierarchical clustering analysis, we used two bootstrap resampling methods implemented in Pvclust: bootstrap probability (BP), the ordinary bootstrap resampling; and the approximately unbiased (AU) [113] probability, from multiscale bootstrap resampling. Both methods were calculated with 10,000 iterations.

## Additional files

Additional file 1: **Table S1.** *T. cruzi* nuclear genome assembly statistics. (XLSX 10 kb)

Additional file 2: **Table S2.** *T. cruzi* CL Brener single-copy gene IDs from the 794 genes recovered from all genome assemblies. (XLSX 20 kb)

Additional file 3: **Table S3.** *T. cruzi* CL Brener single-copy gene IDs from the 701 genes used to estimate nuclear genomic Maximum Likelihood phylogeny. (XLSX 19 kb)

Additional file 4: **Figure S1.** Maxicircle heterozygous SNPs. To test for evidences of mitochondrial heteroplasmy, we evaluated the occurrence of heterozygous SNPs in the whole maxicircle sequence of all seven TcII field isolates and three Y clones. **A)** Total heterozygous SNP count in the maxicircle sequence. **B)** SNPs localized in the mitochondrial coding genes. **C)** SNPs distribution throughout the maxicircle sequence. In each box, the blue lines represent SNP positions, while the black line below corresponds to the whole maxicircle sequence, from 0 to 22,292 kb. In this line, each coding gene is represented by a black box, and the repetitive region is represented by a red box. (DOCX 332 kb)

Additional file 5: **Figure S2.** Boxplot of the predicted ploidy of *T. cruzi* TcII field isolates. The predicted ploidy of each chromosome from the *T. cruzi* field isolates S11, S15, S154a, S162a, S23b, S44a and S92a using as a reference the 41 CL Brener chromosome sequences, was estimated based on the median coverage of all *T. cruzi* genes, excluding those belonging to the largest multigene families, and represented in boxplots. In this image, the predicted ploidy of each of the 41 chromosomes is represented by the median, first and third quartile, as well as maximum and minimum values. **(A)** Representation by strain. In this image, each quadrant corresponds to a TcII strain, containing the predicted ploidy of all 41 chromosomes. **(B)** Representation by chromosome. In this image, each quadrant corresponds to a chromosome, comprising the predicted ploidy of this chromosome in all seven TcII evaluated strains. (PPTX 4680 kb)

Additional file 6: **Table S4.** Ploidy estimations and statistic validation of all 41 chromosomes of the seven TcII field isolates, S11, S15, S154a, S162a, S23b, S44a and S92a. The mean, median and standard deviation of the predicted ploidy of each chromosome of each strain, based on the coverage of all genes in a given chromosome is shown. The evaluation if the predicted ploidy of each chromosome was lower than 1; 1.5 or higher than 2; 2.5; 3; 3.5; 4; 4.5 or 5 was performed based on Mann-Whitney-Wilcoxon tests, with one-way analysis of variance and a significance of *p* < 0.05, using R. Significant values are highlighted in red. (XLSX 46 kb)

Additional file 7: **Figure S3.** Read Depth Coverage of the chromosome 11 in the Y strain and clones. In this picture, the blue lines correspond to the normalized RDC of each position of the chromosome 11, estimated by the ratio between the RDC and the genome coverage. The red line corresponds to the 248 kb position in the chromosome. Below, the protein-coding genes are depicted as rectangles drawn as proportional to their length, and their coding strand is indicated by their position above (top strand) or below (bottom strand) the central line. Cyan and black rectangles represent multigene families and hypothetical/housekeeping genes, respectively. The initial 248-kb in this chromosome had a smaller RDC when compared to remaining sequence in the Y strain as well as in all three Y clones evaluated. (DOCX 226 kb)

Additional file 8: **Table S5.** *T. cruzi* read libraries description. (DOCX 14 kb)

Additional file 9: **Table S6.** Links to download *T. cruzi* reference genomes. (DOCX 12 kb)

Additional file 10: **Table S7.** NCBI accession numbers of the *T. cruzi* genomes and maxicircle assemblies. (DOCX 14 kb)

Additional file 11: **Figure S4.** Competitive mapping of the mitochondrial reads to the three available maxicircle templates. The percentage of mitochondrial genome reads from the 16 *T. cruzi* read libraries that mapped preferentially with each of the maxicircle sequence templates, Sylvio (TcI), Esmeraldo (TcII) and CL Brener (TcVI with mitochondria sequence derived from TcIII) is shown. The TcI strains mapped preferentially with the Sylvio template, while TcII strains mapped preferentially with the Y strain and the TcIII, V and VI strains mapped preferentially with the CL Brener maxicircle sequence. (DOCX 235 kb)

Additional file 12: **Figure S5.** Methodology for *T. cruzi* CCNV estimations. **(A)** The CCNV estimations were performed using the median coverage of all *T. cruzi* genes, excluding those belonging to the largest multigene families in each one of the CL Brener 41 putative chromosomes as an estimate of its chromosome copy number. In brief, the median RDC of the selected genes in each of the 41 CL Brener chromosomes were generated by PERL scripts and normalized by the genome coverage. The genome coverage was estimated as the mean RDC of all single-copy genes in all chromosomes for each strain. **(B)** Heterozygous SNPs between the CL Brener chromosome and the mapped reads for the *T. cruzi* stains were obtained from the filtered SAMtools mpileup results. To be considered as a reliable SNP, the position RDC must be at least 10, with 5 reads supporting each variant. For each chromosome, the proportion of the alleles in each predicted heterozygous site was obtained and rounded to the second place. Base frequencies were rounded in ten categories, ranging from 0.01 to 1.00, and an approximate distribution of base frequencies for each chromosome was plotted in R. Disomic chromosomes have a peak in 0.50, while trisomic chromosomes have peaks in 0.33 and 0.66. Tetrasomic chromosomes have combination of peaks of 0.20, 0.80 and 0.50. (DOCX 120 kb)

Additional file 13: **Table S8.** List of genes used to estimate the chromosomal ploidy of the seven TcII field isolates, after the exclusion of genes with outlier coverages based on iterative Grubbs’ tests. Each isolate, S11, S154a, S162a, S15, S92a, S23b and S44a is represented in a different sheet. (XLSX 1150 kb)
