# Supplementary material for: Whole genome sequencing of Trypanosoma cruzi field isolates reveals extensive genomic variability and complex aneuploidy patterns within TcII DTU
Source: BMC Genomics. 2018 Nov 13;19:816. doi: 10.1186/s12864-018-5198-4 (PMC6234542; doi:10.1186/s12864-018-5198-4)
Supplement: Supplementary file 4 — Figure S1. Maxicircle heterozygous SNPs. To test for evidences of mitochondrial heteroplasmy, we evaluated the occurrence of heterozygous SNPs in the whole maxicircle sequence of all seven TcII field isolates and three Y clones. A) Total heterozygous SNP count in the maxicircle sequence. B) SNPs localized in the mitochondrial coding genes. C) SNPs distribution throughout the maxicircle sequence. In each box, the blue lines represent SNP positions, while the black line below corresponds to the whole maxicircle sequence, from 0 to 22,292 kb. In this line, each coding gene is represented by a black box, and the repetitive region is represented by a red box. (DOCX 332 kb) [file 12864_2018_5198_MOESM4_ESM.docx]

**
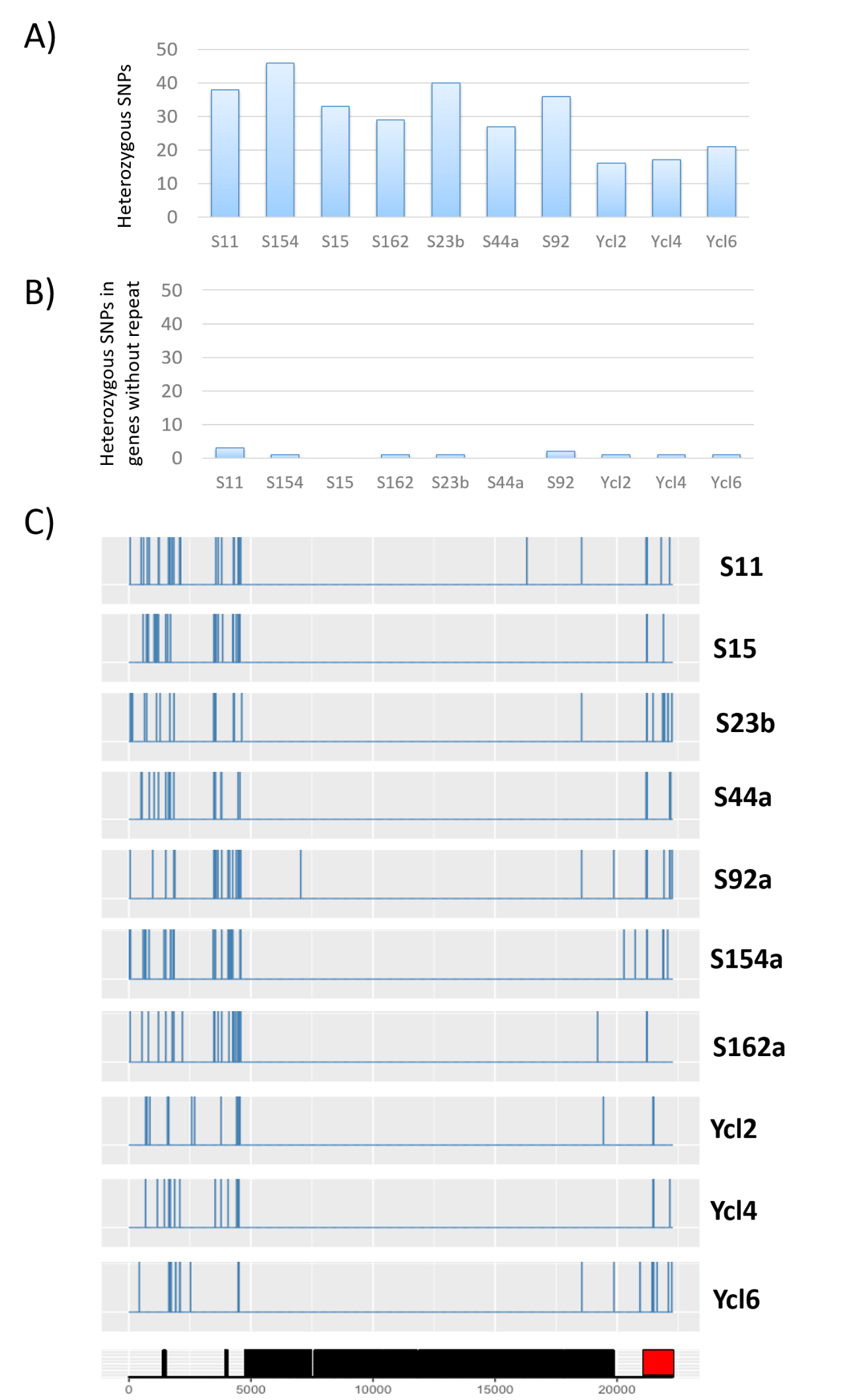
**

**Supplementary Figure 1: Maxicircle heterozygous SNPs**. To test for evidences of mitochondrial heteroplasmy, we evaluated the occurrence of heterozygous SNPs in the whole maxicircle sequence of all seven TcII field isolates and three Y clones. **A)** Total heterozygous SNP count in the maxicircle sequence. B**)** SNPs localized in the mitochondrial coding genes. **C)** SNPs distribution throughout the maxicircle sequence. In each box, the blue lines represent SNP positions, while the black line below corresponds to the whole maxicircle sequence, from 0 to 22,292 kb. In this line, each coding gene is represented by a black box, and the repetitive region is represented by a red box.
