# Supplementary material for: Whole genome sequencing of Trypanosoma cruzi field isolates reveals extensive genomic variability and complex aneuploidy patterns within TcII DTU
Source: BMC Genomics. 2018 Nov 13;19:816. doi: 10.1186/s12864-018-5198-4 (PMC6234542; doi:10.1186/s12864-018-5198-4)
Supplement: Supplementary file 5 — Figure S2. Boxplot of the predicted ploidy of T. cruzi TcII field isolates. The predicted ploidy of each chromosome from the T. cruzi field isolates S11, S15, S154a, S162a, S23b, S44a and S92a using as a reference the 41 CL Brener chromosome sequences, was estimated based on the median coverage of all T. cruzi genes, excluding those belonging to the largest multigene families, and represented in boxplots. In this image, the predicted ploidy of each of the 41 chromosomes is represented by the median, first and third quartile, as well as maximum and minimum values. (A) Representation by strain. In this image, each quadrant corresponds to a TcII strain, containing the predicted ploidy of all 41 chromosomes. (B) Representation by chromosome. In this image, each quadrant corresponds to a chromosome, comprising the predicted ploidy of this chromosome in all seven TcII evaluated strains. (PPTX 4680 kb) [file 12864_2018_5198_MOESM5_ESM.pptx]

## Slide 1
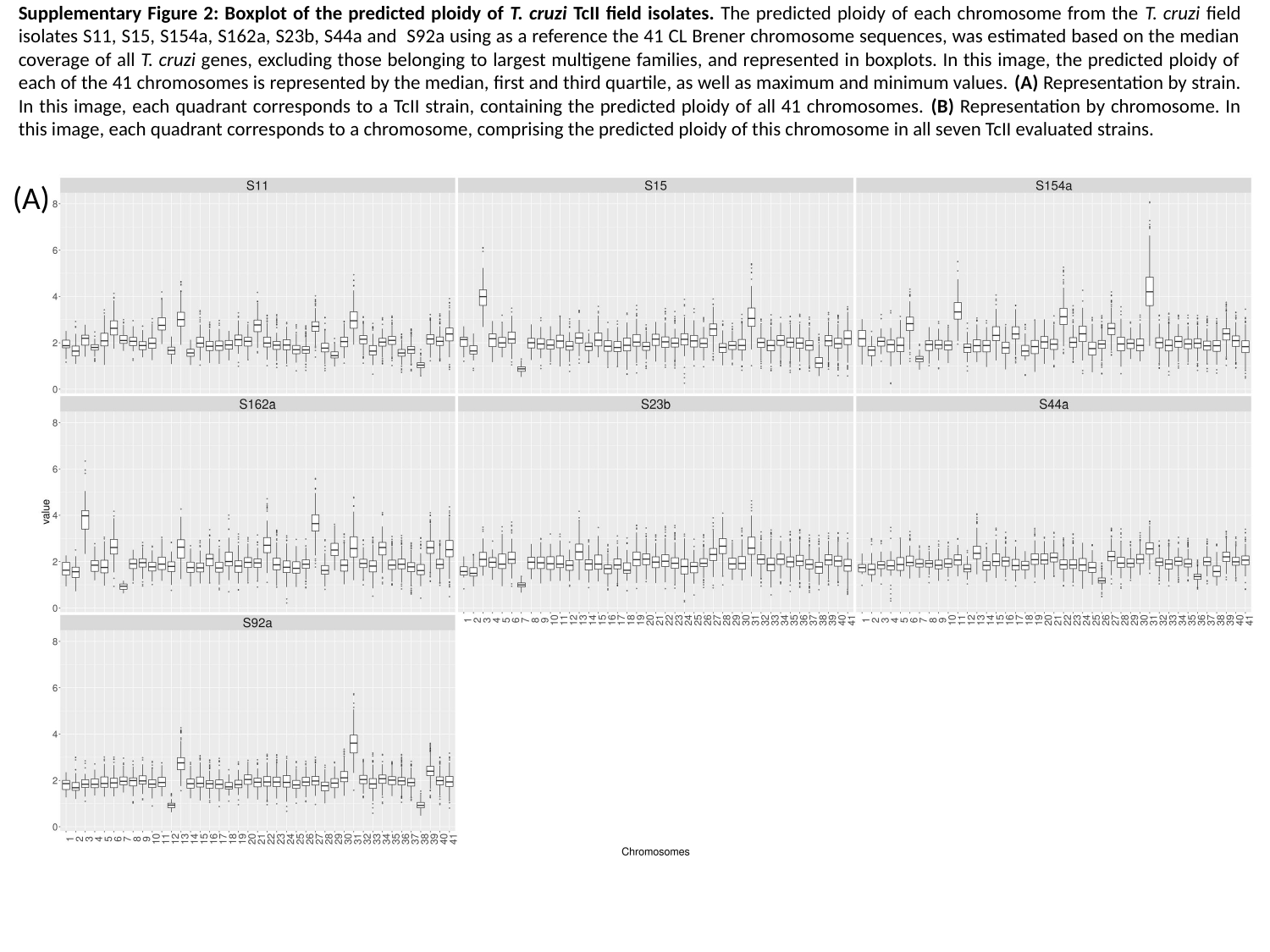

Supplementary Figure 2: Boxplot of the predicted ploidy of T. cruzi TcII field isolates. The predicted ploidy of each chromosome from the T. cruzi field isolates S11, S15, S154a, S162a, S23b, S44a and S92a using as a reference the 41 CL Brener chromosome sequences, was estimated based on the median coverage of all T. cruzi genes, excluding those belonging to largest multigene families, and represented in boxplots. In this image, the predicted ploidy of each of the 41 chromosomes is represented by the median, first and third quartile, as well as maximum and minimum values. (A) Representation by strain. In this image, each quadrant corresponds to a TcII strain, containing the predicted ploidy of all 41 chromosomes. (B) Representation by chromosome. In this image, each quadrant corresponds to a chromosome, comprising the predicted ploidy of this chromosome in all seven TcII evaluated strains.
(A)

## Slide 2
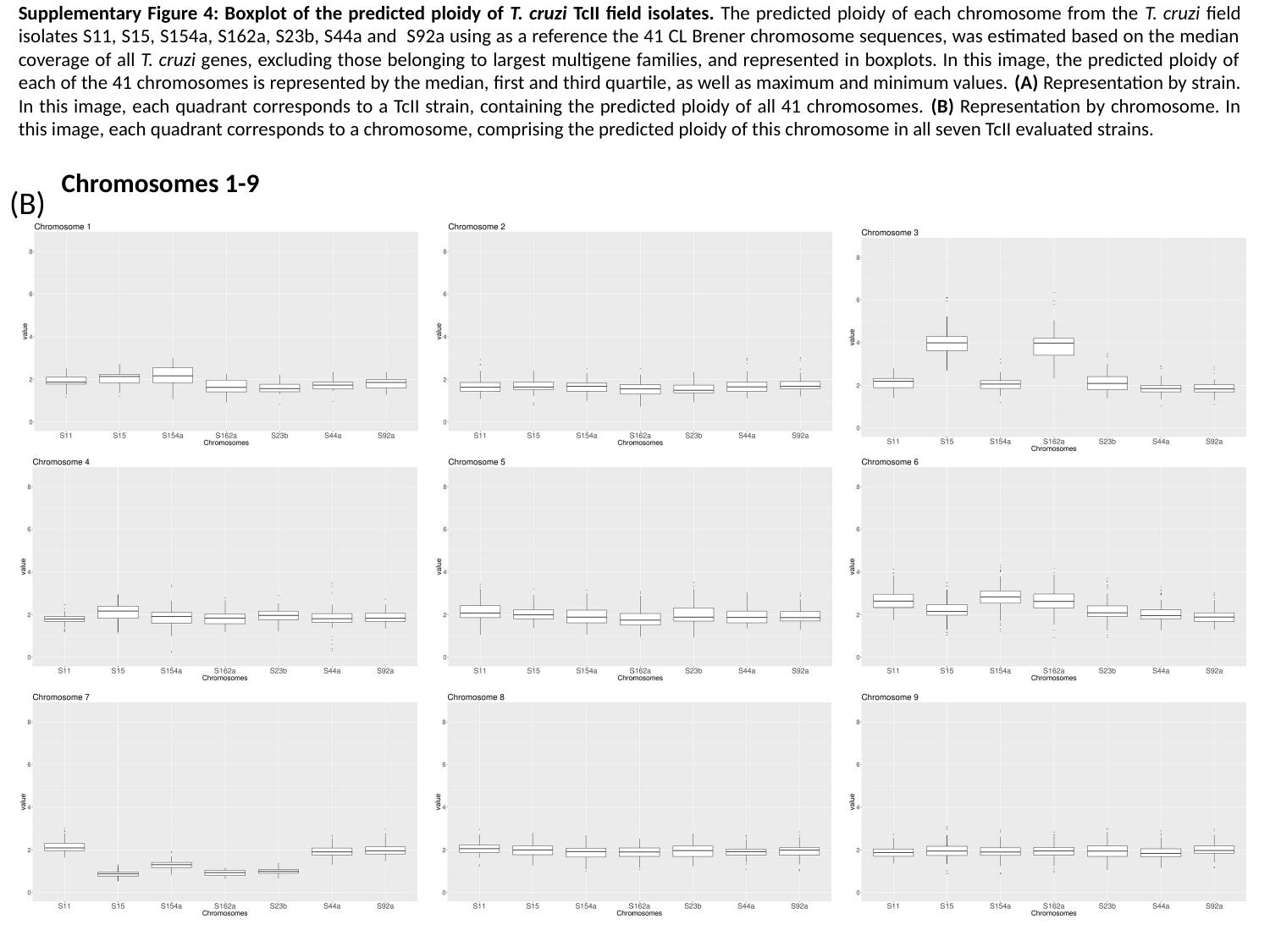

Supplementary Figure 4: Boxplot of the predicted ploidy of T. cruzi TcII field isolates. The predicted ploidy of each chromosome from the T. cruzi field isolates S11, S15, S154a, S162a, S23b, S44a and S92a using as a reference the 41 CL Brener chromosome sequences, was estimated based on the median coverage of all T. cruzi genes, excluding those belonging to largest multigene families, and represented in boxplots. In this image, the predicted ploidy of each of the 41 chromosomes is represented by the median, first and third quartile, as well as maximum and minimum values. (A) Representation by strain. In this image, each quadrant corresponds to a TcII strain, containing the predicted ploidy of all 41 chromosomes. (B) Representation by chromosome. In this image, each quadrant corresponds to a chromosome, comprising the predicted ploidy of this chromosome in all seven TcII evaluated strains.
Chromosomes 1-9
(B)

## Slide 3
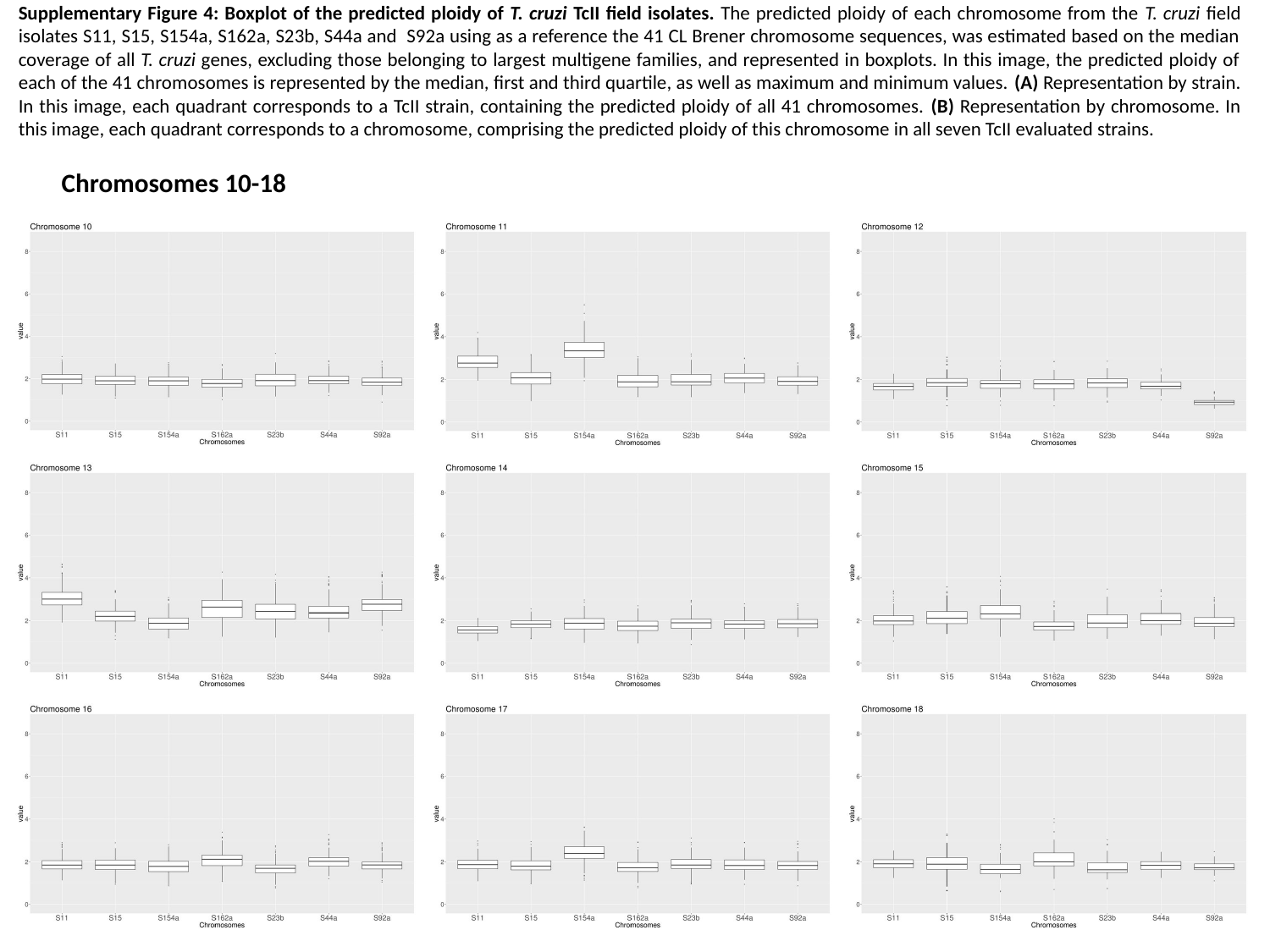

Supplementary Figure 4: Boxplot of the predicted ploidy of T. cruzi TcII field isolates. The predicted ploidy of each chromosome from the T. cruzi field isolates S11, S15, S154a, S162a, S23b, S44a and S92a using as a reference the 41 CL Brener chromosome sequences, was estimated based on the median coverage of all T. cruzi genes, excluding those belonging to largest multigene families, and represented in boxplots. In this image, the predicted ploidy of each of the 41 chromosomes is represented by the median, first and third quartile, as well as maximum and minimum values. (A) Representation by strain. In this image, each quadrant corresponds to a TcII strain, containing the predicted ploidy of all 41 chromosomes. (B) Representation by chromosome. In this image, each quadrant corresponds to a chromosome, comprising the predicted ploidy of this chromosome in all seven TcII evaluated strains.
Chromosomes 10-18

## Slide 4
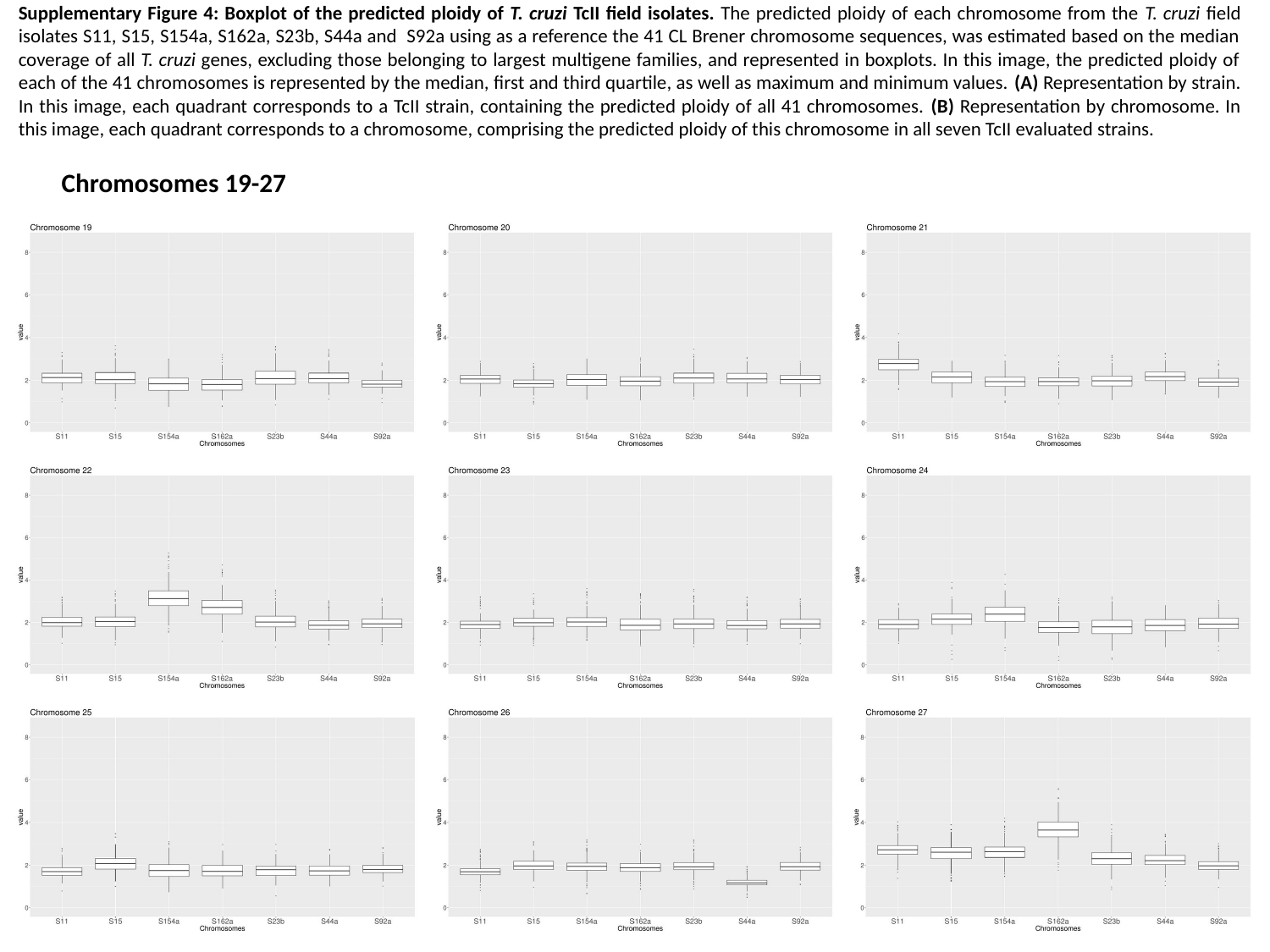

Supplementary Figure 4: Boxplot of the predicted ploidy of T. cruzi TcII field isolates. The predicted ploidy of each chromosome from the T. cruzi field isolates S11, S15, S154a, S162a, S23b, S44a and S92a using as a reference the 41 CL Brener chromosome sequences, was estimated based on the median coverage of all T. cruzi genes, excluding those belonging to largest multigene families, and represented in boxplots. In this image, the predicted ploidy of each of the 41 chromosomes is represented by the median, first and third quartile, as well as maximum and minimum values. (A) Representation by strain. In this image, each quadrant corresponds to a TcII strain, containing the predicted ploidy of all 41 chromosomes. (B) Representation by chromosome. In this image, each quadrant corresponds to a chromosome, comprising the predicted ploidy of this chromosome in all seven TcII evaluated strains.
Chromosomes 19-27

## Slide 5
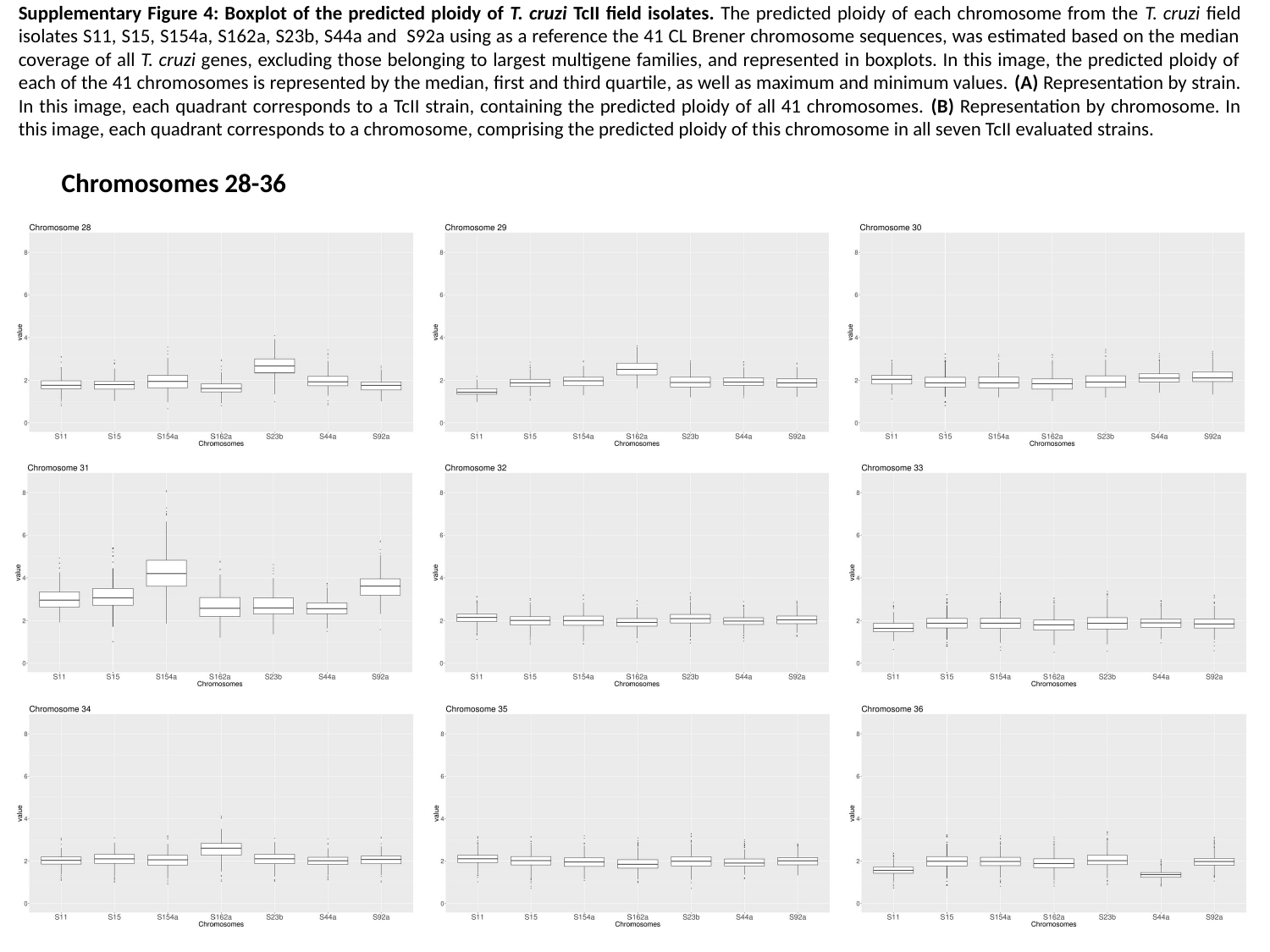

Supplementary Figure 4: Boxplot of the predicted ploidy of T. cruzi TcII field isolates. The predicted ploidy of each chromosome from the T. cruzi field isolates S11, S15, S154a, S162a, S23b, S44a and S92a using as a reference the 41 CL Brener chromosome sequences, was estimated based on the median coverage of all T. cruzi genes, excluding those belonging to largest multigene families, and represented in boxplots. In this image, the predicted ploidy of each of the 41 chromosomes is represented by the median, first and third quartile, as well as maximum and minimum values. (A) Representation by strain. In this image, each quadrant corresponds to a TcII strain, containing the predicted ploidy of all 41 chromosomes. (B) Representation by chromosome. In this image, each quadrant corresponds to a chromosome, comprising the predicted ploidy of this chromosome in all seven TcII evaluated strains.
Chromosomes 28-36

## Slide 6
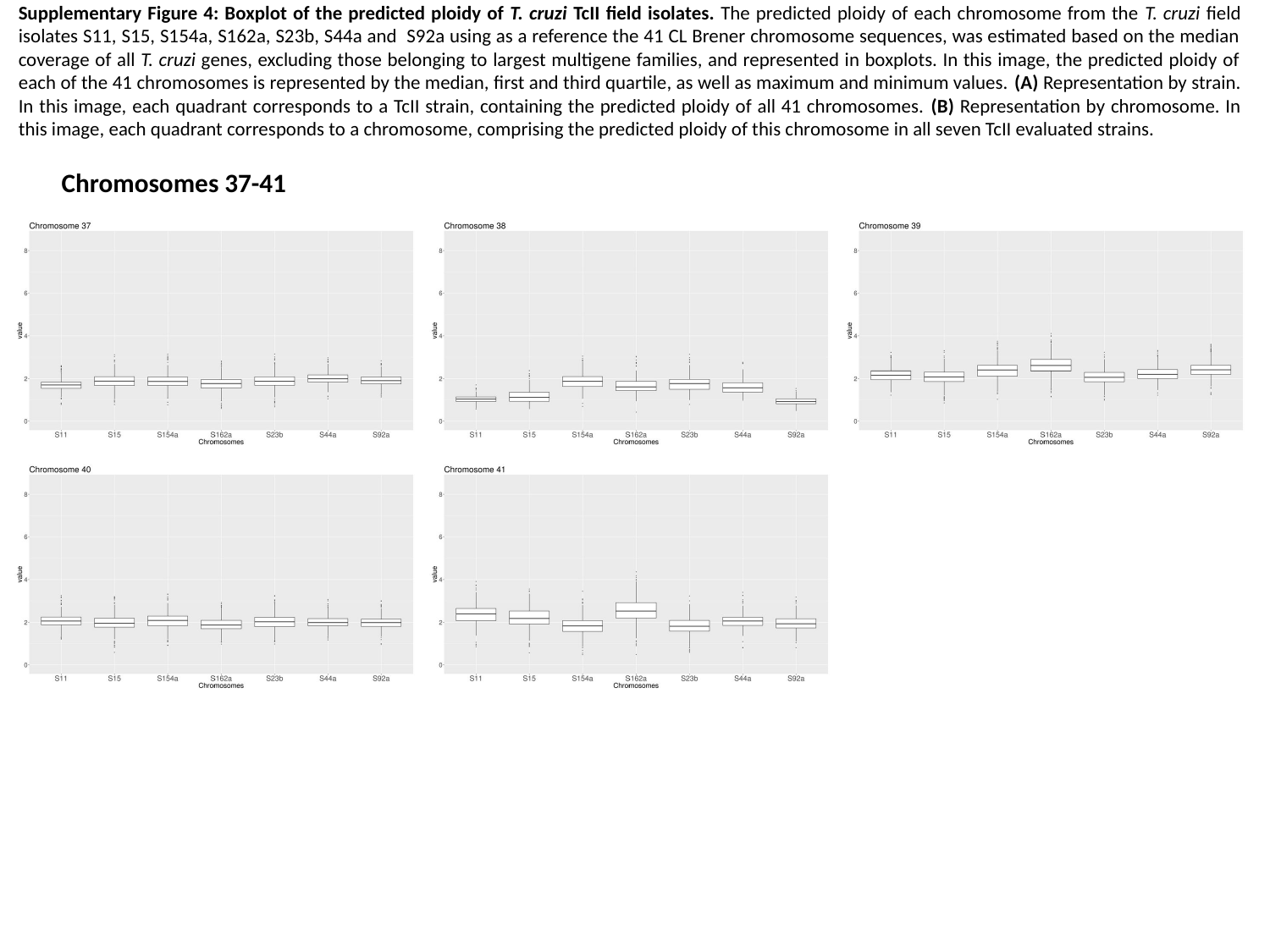

Supplementary Figure 4: Boxplot of the predicted ploidy of T. cruzi TcII field isolates. The predicted ploidy of each chromosome from the T. cruzi field isolates S11, S15, S154a, S162a, S23b, S44a and S92a using as a reference the 41 CL Brener chromosome sequences, was estimated based on the median coverage of all T. cruzi genes, excluding those belonging to largest multigene families, and represented in boxplots. In this image, the predicted ploidy of each of the 41 chromosomes is represented by the median, first and third quartile, as well as maximum and minimum values. (A) Representation by strain. In this image, each quadrant corresponds to a TcII strain, containing the predicted ploidy of all 41 chromosomes. (B) Representation by chromosome. In this image, each quadrant corresponds to a chromosome, comprising the predicted ploidy of this chromosome in all seven TcII evaluated strains.
Chromosomes 37-41
