# Supplementary material for: Whole genome sequencing of Trypanosoma cruzi field isolates reveals extensive genomic variability and complex aneuploidy patterns within TcII DTU
Source: BMC Genomics. 2018 Nov 13;19:816. doi: 10.1186/s12864-018-5198-4 (PMC6234542; doi:10.1186/s12864-018-5198-4)
Supplement: Supplementary file 7 — Figure S3. Read Depth Coverage of the chromosome 11 in the Y strain and clones. In this picture, the blue lines correspond to the normalized RDC of each position of the chromosome 11, estimated by the ratio between the RDC and the genome coverage. The red line corresponds to the 248 kb position in the chromosome. Below, the protein-coding genes are depicted as rectangles drawn as proportional to their length, and their coding strand is indicated by their position above (top strand) or below (bottom strand) the central line. Cyan and black rectangles represent multigene families and hypothetical/housekeeping genes, respectively. The initial 248-kb in this chromosome had a smaller RDC when compared to remaining sequence in the Y strain as well as in all three Y clones evaluated. (DOCX 226 kb) [file 12864_2018_5198_MOESM7_ESM.docx]

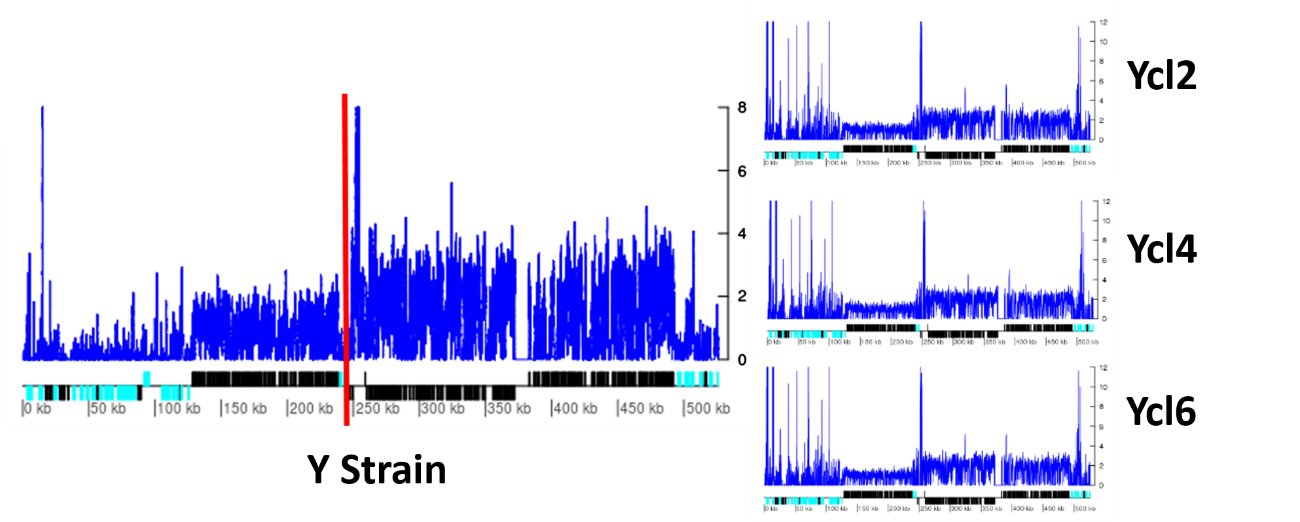


**Supplementary Figure 3: Read Depth Coverage of the chromosome 11 in the Y strain and clones.** In this picture, the blue lines correspond to the normalized RDC of each position of the chromosome 11, estimated by the ratio between the RDC and the genome coverage. The red line corresponds to the 248kb position in the chromosome. Below, the protein-coding genes are depicted as rectangles drawn as proportional to their length, and their coding strand is indicated by their position above (top strand) or below (bottom strand) the central line. Cyan and black rectangles represent multigene families and hypothetical/housekeeping genes, respectively. The initial 248-kb in this chromosome had a smaller RDC when compared to remaining sequence in the Y strain as well as in all three Y clones evaluated.
