# Supplementary material for: Whole genome sequencing of Trypanosoma cruzi field isolates reveals extensive genomic variability and complex aneuploidy patterns within TcII DTU
Source: BMC Genomics. 2018 Nov 13;19:816. doi: 10.1186/s12864-018-5198-4 (PMC6234542; doi:10.1186/s12864-018-5198-4)
Supplement: Supplementary file 8 — Table S5. T. cruzi read libraries description. (DOCX 14 kb) [file 12864_2018_5198_MOESM8_ESM.docx]

**Supplementary Table 5: *T. cruzi* read libraries description.**

| **Sample ID** | **Sequencing**  **Platform** | **DTU** | **Accession Number** | **Patient Clinical Stage** | **City of Isolation** |
| --- | --- | --- | --- | --- | --- |
| Arequipa | 454/Ion Torrent | TcI | SRS838181 | Nd | Arequipa/Peru |
| Colombiana | 454/Ion Torrent | TcI | SRS841912 | Nd | Colombia |
| Sylvio | 454 | TcI | - | Nd | Pará/Brazil |
| S11 | Illumina | TcII | SRR6357362 | Indetermined | Itaipé/Brazil |
| S15 | Illumina | TcII | SRR6357361 | Indetermined | Felixlândia/Brazil |
| S154a | Illumina | TcII | SRR6357354 | Indetermined | Itaipé/Brazil |
| S162a | Illumina | TcII | SRR6357360 | indetermined | Congonhas do  Norte/Brazil |
| S23b | Illumina | TcII | SRR6357359 | Cardiac | Porteirinha/Brazil |
| S44a | Illumina | TcII | SRR6357358 | Indetermined | Turmalina/Brazil |
| S92a | Illumina | TcII | SRR6357357 | Cardiac | Teófilo Otoni/Brazil |
| Esmeraldo | 454/Illumina | TcII | SRR833799/SRR833800/ SRR058517/SRR058509/ SRR058520/SRR058518/  SRR058519/ SRR058515/ SRR058516/SRR058513/  SRR058514/SRR058510/ SRR058511/SRR058512 | Nd | Bahia/Brazil |
| Y-population | 454/Ion Torrent | TcII | SRS842149 | Nd | São Paulo/Brazil |
| Y-cl2 | Illumina | TcII | SRR6357356 | Nd | São Paulo/Brazil |
| Y-cl4 | Illumina | TcII | SRR6357355 | Nd | São Paulo/Brazil |
| Y-cl6 | Illumina | TcII | SRR6357364 | Nd | São Paulo/Brazil |
| 231 | Illumina | TcIII | [ERR864236](https://trace.ncbi.nlm.nih.gov/Traces/sra/?run=ERR864236) | Indetermined | Minas Gerais/Brazil |
| 9280 | Illumina | TcV | [SRR1996502](https://trace.ncbi.nlm.nih.gov/Traces/sra/?run=SRR1996502)/ [SRR1996492](https://trace.ncbi.nlm.nih.gov/Traces/sra/?run=SRR1996492)/ [SRR1996496](https://trace.ncbi.nlm.nih.gov/Traces/sra/?run=SRR1996496)/ [SRR1996493](https://trace.ncbi.nlm.nih.gov/Traces/sra/?run=SRR1996493)/  [SRR1996497](https://trace.ncbi.nlm.nih.gov/Traces/sra/?run=SRR1996497) | Nd | Santa Cruz/Bolivia |
| Tulahuen | Illumina | TcVI | SRX268895/ SRX268893 | Nd | Tulahuen/Chile |
| CL Brener | Illumina | TcVI | SRR6357363 | Nd | Rio Grande do Sul/Brazil |

Nd=Non determined.
