# Supplementary material for: Whole genome sequencing of Trypanosoma cruzi field isolates reveals extensive genomic variability and complex aneuploidy patterns within TcII DTU
Source: BMC Genomics. 2018 Nov 13;19:816. doi: 10.1186/s12864-018-5198-4 (PMC6234542; doi:10.1186/s12864-018-5198-4)
Supplement: Supplementary file 9 — Table S6. Links to download T. cruzi reference genomes. (DOCX 12 kb) [file 12864_2018_5198_MOESM9_ESM.docx]

**Supplementary Table 6: Links to download *T. cruzi* reference genomes.**

| **Genome** | **Link** |
| --- | --- |
| **Esmeraldo** | <http://www.ebi.ac.uk/ena/data/view/ANOX01000001-ANOX01020187> |
| **231** | <https://www.ncbi.nlm.nih.gov/assembly/GCA_900252365.1/> |
| **Sylvio** | <http://tritrypdb.org/common/downloads/release-29/TcruziSylvioX10-1/fasta/data/TriTrypDB-29_TcruziSylvioX10-1_Genome.fasta> |
| **CL Brener Esmeraldo-like haplotype** | <http://tritrypdb.org/common/downloads/release-26/TcruziCLBrenerEsmeraldo-like/fasta/data/TriTrypDB-26_TcruziCLBrenerEsmeraldo-like_Genome.fasta> |
| **CL Brener Non-Esmeraldo haplotype** | <http://tritrypdb.org/common/downloads/release-26/TcruziCLBrenerNon-Esmeraldo-like/fasta/data/TriTrypDB-26_TcruziCLBrenerNon-Esmeraldo-like_Genome.fasta> |
