# Supplementary material for: Whole genome sequencing of Trypanosoma cruzi field isolates reveals extensive genomic variability and complex aneuploidy patterns within TcII DTU
Source: BMC Genomics. 2018 Nov 13;19:816. doi: 10.1186/s12864-018-5198-4 (PMC6234542; doi:10.1186/s12864-018-5198-4)
Supplement: Supplementary file 10 — Table S7. NCBI accession numbers of the T. cruzi genomes and maxicircle assemblies. (DOCX 14 kb) [file 12864_2018_5198_MOESM10_ESM.docx]

**Supplementary Table 7:** NCBI accession numbers of the *T. cruzi* genomes and maxicircle assemblies.

| **Sample id** | **Nuclear NCBI Accession ID** | **Maxicircle NCBI Accession ID** |
| --- | --- | --- |
| Arequipa | PYLF00000000 | MH144187 |
| Colombiana | PYLG00000000 | MH144189 |
| S11 | PYKV00000000 | MH144190 |
| S15 | PYLE00000000 | MH144192 |
| S154a | PYLD00000000 | MH144191 |
| S162a | PYLC00000000 | MH144193 |
| S23b | PYLB00000000 | MH144194 |
| S44a | PYLA00000000 | MH144195 |
| S92a | PYKZ00000000 | MH144196 |
| Y-cl2 | PYKY00000000 | MH144197 |
| Y-cl4 | PYKX00000000 | MH144198 |
| Y-cl6 | PYKW00000000 | MH144199 |
| Y-population | PYLH00000000 | MH144200 |
