# Supplementary material for: Whole genome sequencing of Trypanosoma cruzi field isolates reveals extensive genomic variability and complex aneuploidy patterns within TcII DTU
Source: BMC Genomics. 2018 Nov 13;19:816. doi: 10.1186/s12864-018-5198-4 (PMC6234542; doi:10.1186/s12864-018-5198-4)
Supplement: Supplementary file 11 — Figure S4. Competitive mapping of the mitochondrial reads to the three available maxicircle templates. The percentage of mitochondrial genome reads from the 16 T. cruzi read libraries that mapped preferentially with each of the maxicircle sequence templates, Sylvio (TcI), Esmeraldo (TcII) and CL Brener (TcVI with mitochondria sequence derived from TcIII) is shown. The TcI strains mapped preferentially with the Sylvio template, while TcII strains mapped preferentially with the Y strain and the TcIII, V and VI strains mapped preferentially with the CL Brener maxicircle sequence. (DOCX 235 kb) [file 12864_2018_5198_MOESM11_ESM.docx]

**
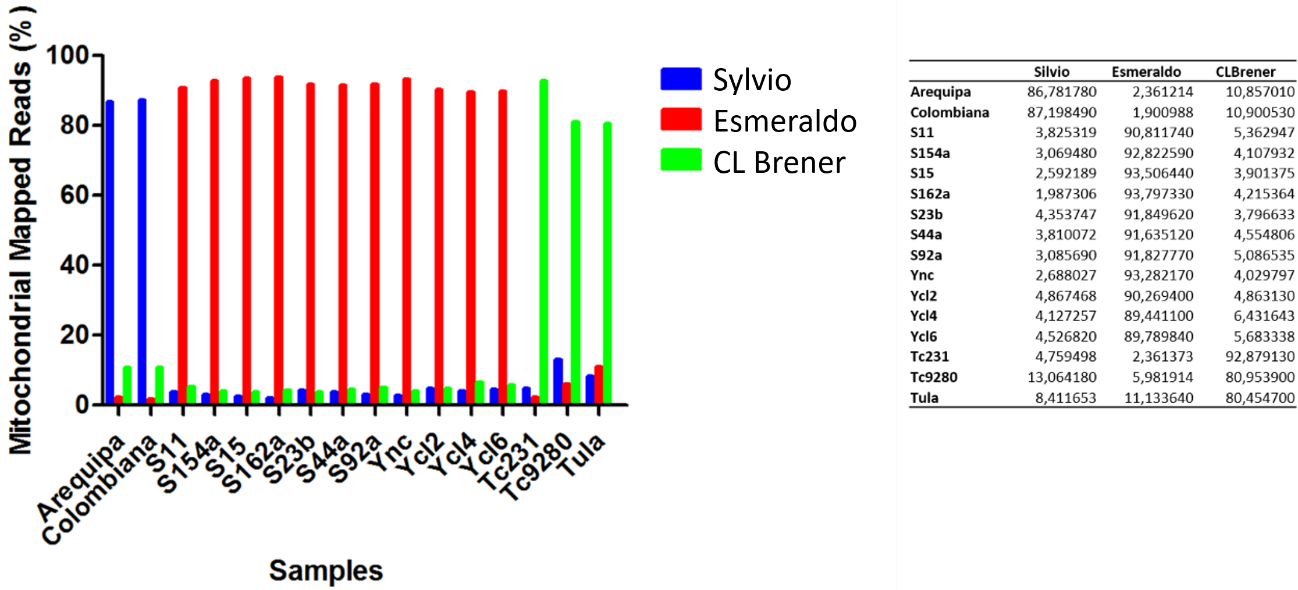
**

**Supplementary Figure 4: Competitive mapping of the mitochondrial reads to the three available maxicircle templates.** The percentage of mitochondrial genome reads from the 16 *T. cruzi* read libraries that mapped preferentially with each of the maxicircle sequence templates, Sylvio (TcI), Esmeraldo (TcII) and CL Brener (TcVI with mitochondria sequence derived from TcIII) is shown. The TcI strains mapped preferentially with the Sylvio template, while TcII strains mapped preferentially with the Y strain and the TcIII, V and VI strains mapped preferentially with the CL Brener maxicircle sequence.
