# Supplementary material for: Whole genome sequencing of Trypanosoma cruzi field isolates reveals extensive genomic variability and complex aneuploidy patterns within TcII DTU
Source: BMC Genomics. 2018 Nov 13;19:816. doi: 10.1186/s12864-018-5198-4 (PMC6234542; doi:10.1186/s12864-018-5198-4)
Supplement: Supplementary file 12 — Figure S5. Methodology for T. cruzi CCNV estimations. (A) The CCNV estimations were performed using the median coverage of all T. cruzi genes, excluding those belonging to the largest multigene families in each one of the CL Brener 41 putative chromosomes as an estimate of its chromosome copy number. In brief, the median RDC of the selected genes in each of the 41 CL Brener chromosomes were generated by PERL scripts and normalized by the genome coverage. The genome coverage was estimated as the mean RDC of all single-copy genes in all chromosomes for each strain. (B) Heterozygous SNPs between the CL Brener chromosome and the mapped reads for the T. cruzi stains were obtained from the filtered SAMtools mpileup results. To be considered as a reliable SNP, the position RDC must be at least 10, with 5 reads supporting each variant. For each chromosome, the proportion of the alleles in each predicted heterozygous site was obtained and rounded to the second place. Base frequencies were rounded in ten categories, ranging from 0.01 to 1.00, and an approximate distribution of base frequencies for each chromosome was plotted in R. Disomic chromosomes have a peak in 0.50, while trisomic chromosomes have peaks in 0.33 and 0.66. Tetrasomic chromosomes have combination of peaks of 0.20, 0.80 and 0.50. (DOCX 120 kb) [file 12864_2018_5198_MOESM12_ESM.docx]

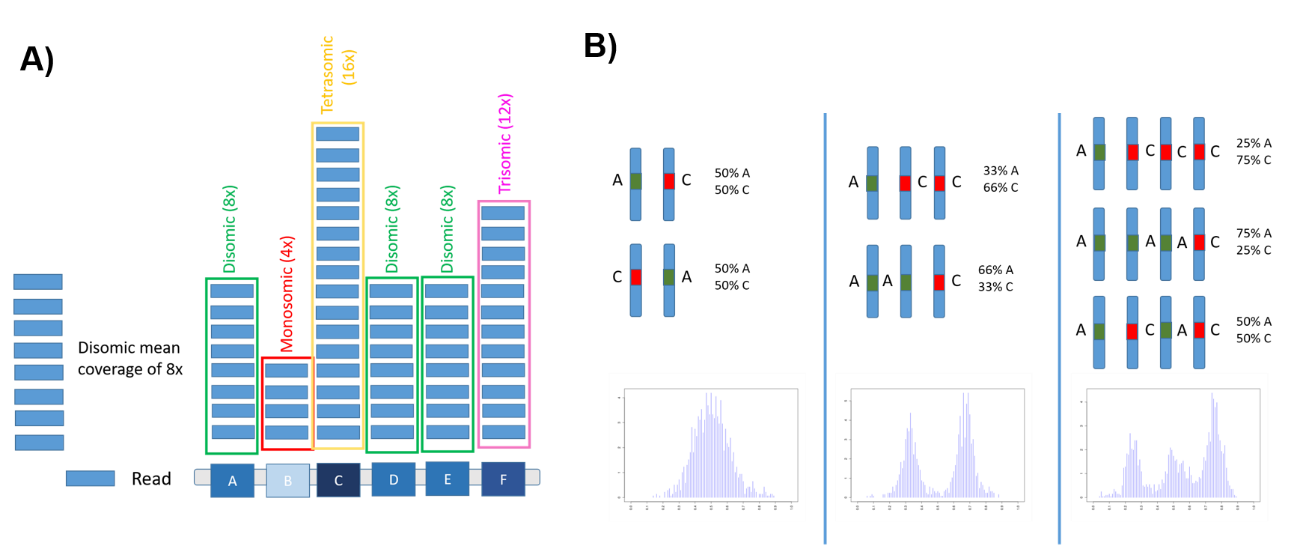


**Supplementary Figure 5: Methodology for *T. cruzi* CCNV estimations.** **(A)** The CCNV estimations were performed using the median coverage of all *T. cruzi* genes, excluding those belonging to the largest multigene families in each one of the CL Brener 41 putative chromosomes as an estimate of its chromosome copy number. In brief, the median RDC of the selected genes in each of the 41 CL Brener chromosomes were generated by PERL scripts and normalized by the genome coverage. The genome coverage was estimated as the mean RDC of all single-copy genes in all chromosomes for each strain. **(B)** Heterozygous SNPs between the CL Brener chromosome and the mapped reads for the *T. cruzi* stains were obtained from the filtered SAMtools mpileup results. To be considered as a reliable SNP, the position RDC must be at least 10, with 5 reads supporting each variant. For each chromosome, the proportion of the alleles in each predicted heterozygous site was obtained and rounded to the second place. Base frequencies were rounded in ten categories, ranging from 0.01 to 1.00, and an approximate distribution of base frequencies for each chromosome was plotted in R. Disomic chromosomes had a peak in 0.50, while trisomic chromosomes had peaks in 0.33 and 0.66. Tetrasomic chromosomes had combination of peaks of 0.20, 0.80 and 0.50.
